# Unraveling the genetic architecture for carbon and nitrogen related traits and leaf hydraulic conductance in soybean using genome-wide association analyses

**DOI:** 10.1186/s12864-019-6170-7

**Published:** 2019-11-06

**Authors:** Clinton J. Steketee, Thomas R. Sinclair, Mandeep K. Riar, William T. Schapaugh, Zenglu Li

**Affiliations:** 1 Institute of Plant Breeding, Genetics, and Genomics and Department of Crop and Soil Sciences, University of Georgia, Athens, GA USA; 2 Department of Crop and Soil Sciences, North Carolina State University, Raleigh, NC USA; 3 Department of Agronomy, Kansas State University, Manhattan, KS USA

**Keywords:** Soybean, *Glycine max*, Drought tolerance, Carbon isotope composition, Nitrogen concentration, Nitrogen isotope composition, Aquaporin, Genome-wide association study (GWAS)

## Abstract

**Background:**

Drought stress is a major limiting factor of soybean [*Glycine max* (L.) Merr.] production around the world. Soybean plants can ameliorate this stress with improved water-saving, sustained N_2_ fixation during water deficits, and/or limited leaf hydraulic conductance. In this study, carbon isotope composition (δ^13^C), which can relate to variation in water-saving capability, was measured. Additionally, nitrogen isotope composition (δ^15^N) and nitrogen concentration that relate to nitrogen fixation were evaluated. Decrease in transpiration rate (DTR) of de-rooted soybean shoots in a silver nitrate (AgNO_3_) solution compared to deionized water under high vapor pressure deficit (VPD) conditions was used as a surrogate measurement for limited leaf hydraulic conductance. A panel of over 200 genetically diverse soybean accessions genotyped with the SoySNP50K iSelect BeadChips was evaluated for the carbon and nitrogen related traits in two field environments (Athens, GA in 2015 and 2016) and for transpiration response to AgNO_3_ in a growth chamber. A multiple loci linear mixed model was implemented in FarmCPU to perform genome-wide association analyses for these traits.

**Results:**

Thirty two, 23, 26, and nine loci for δ^13^C, δ^15^N, nitrogen concentration, and transpiration response to AgNO_3_, respectively, were significantly associated with these traits. Candidate genes that relate to drought stress tolerance enhancement or response were identified near certain loci that could be targets for improving and understanding these traits. Soybean accessions with favorable breeding values were also identified. Low correlations were observed between many of the traits and the genetic loci associated with each trait were largely unique, indicating that these drought tolerance related traits are governed by different genetic loci.

**Conclusions:**

The genomic regions and germplasm identified in this study can be used by breeders to understand the genetic architecture for these traits and to improve soybean drought tolerance. Phenotyping resources needed, trait heritability, and relationship to the target environment should be considered before deciding which of these traits to ultimately employ in a specific breeding program. Potential marker-assisted selection efforts could focus on loci which explain the greatest amount of phenotypic variation for each trait, but may be challenging due to the quantitative nature of these traits.

## Background

Soybean [*Glycine max* (L.) Merr.] seeds are an important source of protein and oil for a range of applications. Drought stress is the most important abiotic factor affecting soybean production, and can cause large decreases in yield [1]. Use of irrigation during drought stress could ameliorate this issue; however, less than 10% of U.S. soybean hectares are irrigated [2]. Therefore, the development of soybean cultivars that can withstand periods of drought stress is necessary to protect yield when water resources are limited.

Certain morphological and physiological traits could reflect the ability of soybean plants to better tolerate drought stress. Carbon isotope composition has been previously identified as a useful screening method to understand photosynthetic tradeoffs and water-saving capabilities of C3 plant species in certain environments [3–7]. C3 plants readily assimilate the ^12^C isotope of carbon in photosynthesis, and therefore discriminate against the heavier ^13^C isotope, which constitutes only around 1% of the atmosphere [4]. Carbon isotope composition can be expressed as either carbon isotope discrimination (Δ^13^C, CID) or carbon isotope ratio (δ^13^C). Carbon isotope composition has been used as an indirect method for selection of genotypes with improved productivity in drought-stressed environments. However, it should be noted that in some cases CID has not been a good indicator for drought tolerance or did not produce consistent genotypic rankings across environments [8–10].

Additionally, previous genome-wide association studies (GWAS) and quantitative trait locus (QTL) mapping studies have identified genomic regions controlling carbon isotope composition in soybean. In one of these studies, 373 diverse maturity group (MG) IV soybean genotypes were grown in four environments and 39 single nucleotide polymorphisms (SNPs) were identified with GWAS that had significant association with δ^13^C in at least two environments [11]. Another study using the same set of accessions and phenotypic data, but with ~ 20,000 additional SNP markers and a different GWAS model, found 54 environment-specific SNPs tagging 46 putative loci for δ^13^C [12]. Previous QTL mapping in soybean identified five loci controlling CID [13].

Soybean is a legume which uses a symbiotic association with bradyrhizobia to fix N_2_ from the atmosphere. This nitrogen fixation provides a supply of nitrogen (N) to the plant that is used for growth and development, as well as providing nitrogen in the crop residue for subsequent crops when soybean is used in a crop rotation. However, symbiotic N_2_ fixation can be affected by limited water availability, and certain soybean genotypes are more sensitive than others in regards to N_2_ fixation during drought stress [14–18]. A previous simulation study that investigated the benefits of altered soybean drought traits found that sustained N_2_ fixation during water deficits had the most consistent and greatest yield advantage compared to four other traits using 50 years of weather data across U.S. soybean growing regions [19].

Using a three-stage screening process, [20] identified eight soybean genotypes with superior N_2_ fixation during water deficits. In addition, PI 471938 has been reported to have tolerant N_2_ fixation as soil dries [21]. Differences in the amount of N present in leaf tissue have previously been used as a way to determine a soybean genotype’s sensitivity to N_2_ fixation during drought conditions, with lower foliar N concentrations having superior fixation during water deficits [14, 17, 18]. This could be due to genotypes with higher plant N concentrations under well-watered conditions being closer to a threshold N level in the plant that can trigger a negative feedback of nitrogen compounds decreasing N_2_ fixation rate. In contrast, genotypes with lower plant N concentrations may continue to fix nitrogen during water deficits due to a lack of this feedback. Four QTLs for foliar N concentration were previously identified on Chr 13, 16, and 17 using a ‘KS4895’ × ‘Jackson’ RIL population [22].

Nitrogen isotope composition (δ^15^N) could be a useful evaluation tool given that ^15^N is present at much greater levels in soil compared to the atmosphere [23–25]. The fraction of ^15^N found in a soybean plant would be decreased if it is actively fixing N_2_ from the atmosphere, and could be an indicator of how much nitrogen fixation is affected by drought stress [26]. A previous association mapping study using 373 soybean genotypes in MG IV found 19 and 17 SNP markers significantly associated with N concentration and the fraction of N derived from the atmosphere (Ndfa), respectively, that were found in at least two of the four environments tested [26].

Leaf hydraulic conductance is defined as the water flux through the leaf per unit water potential driving force, and is a measure of how readily water flows through the leaf [27]. Limited leaf hydraulic conductance is a trait related to soybean drought tolerance that results in conserved soil moisture for use during subsequent water deficits. According to previous research, decreased hydraulic conductance allows certain soybean plants, namely PI 416937, to conserve soil water and express a slow canopy-wilting phenotype in the field after extended periods with little to no precipitation [28]. Additionally, it was hypothesized that differences in hydraulic conductance were a result of different populations of aquaporins, water-conducting membrane proteins that are involved in water movement through cell membranes. It was suggested that these aquaporin populations could be differentiated due to differences in sensitivity to exposure to certain chemical inhibitors [29]. Subjecting de-rooted soybean shoots to a silver nitrate (AgNO_3_) solution under high vapor pressure deficit (VPD) conditions resulted in some genotypes expressing a decreased transpiration rate, and it was hypothesized that this decrease in transpiration was a result of silver ions blocking silver-sensitive aquaporins. PI 416937, a slow-wilting genotype with low hydraulic conductance, exhibited an insensitivity to silver nitrate by not decreasing its transpiration rate when subjected to the inhibitor solution [30]. Given the possible relationship of the transpiration response to silver nitrate and hydraulic conductance, soybean genotypes could be characterized using this procedure to potentially differentiate aquaporin populations and identify drought tolerant germplasm. A previous QTL mapping study identified four QTLs explaining 17.7 to 24.7% of the phenotypic variation for the limited leaf hydraulic conductance trait using transpiration response to silver nitrate as the measurement for the trait [31].

In this study, a genetically diverse panel of over 200 soybean genotypes was evaluated for δ^13^C, δ^15^N, and foliar nitrogen concentration from leaf samples collected in two field environments. Additionally, this panel was evaluated for transpiration response to silver nitrate under high VPD conditions in a growth chamber. The objectives of this study were to identify genomic regions controlling these traits using genome-wide association analyses, validate genomic loci for these traits across environments or studies, and identify genotypes in the panel which have favorable breeding values for these traits.

## Results

### δ^13^C, δ^15^N, and N concentration

**Fig. 1:**
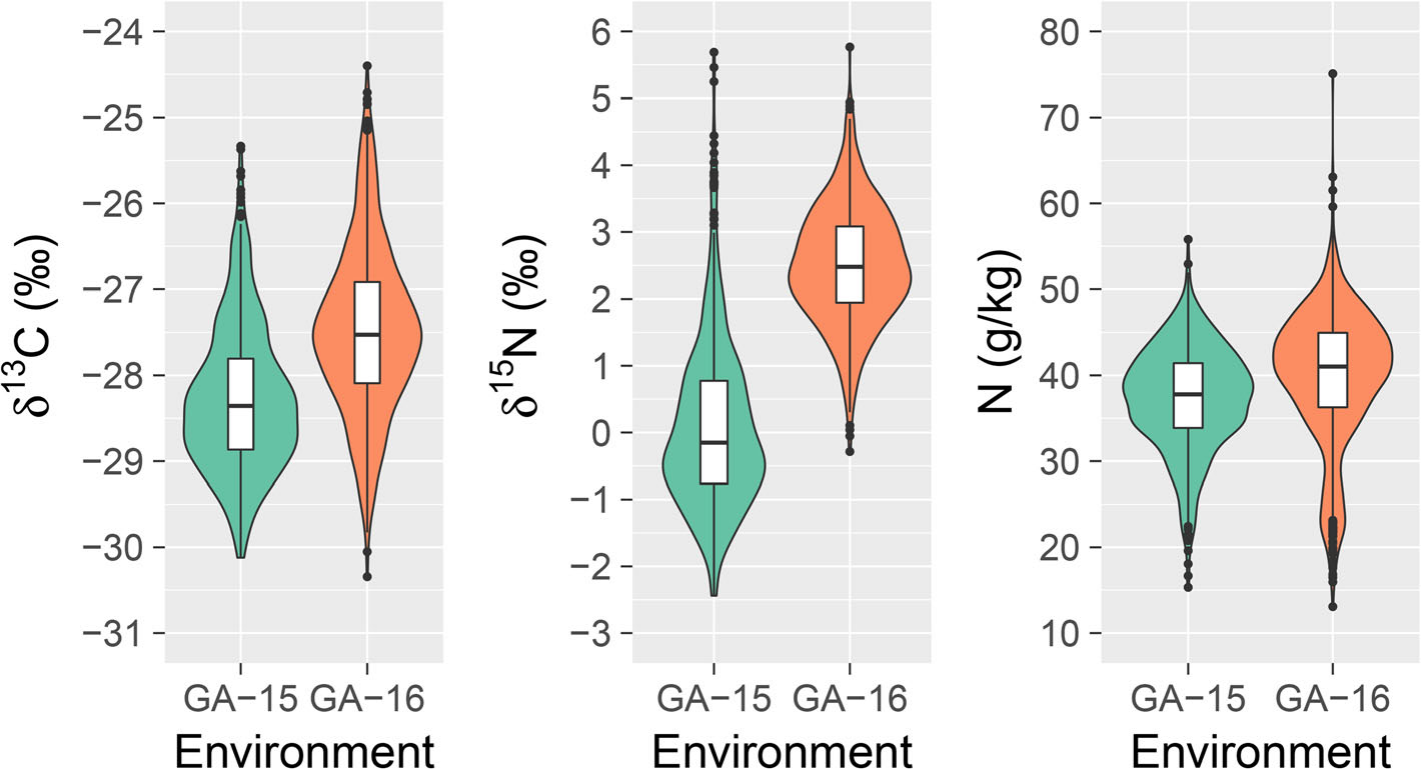
Violin plots with boxplots inside for carbon and nitrogen related traits. Individual plot data evaluated in two environments with association panel are shown

Carbon isotope composition (δ^13^C), nitrogen isotope composition (δ^15^N), and foliar nitrogen (N) concentration were evaluated in two field environments (GA-15 and GA-16). Based on the analyses of variance (ANOVA), genotypes, environments, and their interaction were statistically significant (*p* < 0.05) for all carbon and nitrogen related traits (Table 1). Genotype mean values within environments of δ^13^C ranged from − 29.97 to − 25.14‰ (Fig. 1), and had a correlation of r = 0.74 between environments. Broad-sense heritability of δ^13^C on an entry-mean basis for each environment was 61% (GA-15), 72% (GA-16), and 62% across both environments (Table 2). δ^15^N had a correlation of r = 0.28 between environments, and ranged from − 1.23 to 4.50‰ based on mean genotype values within environments (Fig. 1). Heritability for δ^15^N was lower than for all other carbon and nitrogen related traits at 24% (GA-15), 40% (GA-16), and 17% across both environments (Both) (Table 2). The range of leaf nitrogen concentrations observed for genotype means within environments was from 16.67 to 55.45 g kg^− 1^, and the correlation between the two environments was r = 0.73. Broad-sense heritability for N concentration was between 63 and 73% (Table 2).

**Table 1:** Summary of analyses of variance (ANOVA) for each trait evaluated

Carbon Isotope Composition (δ^13^C)				Nitrogen Isotope Composition (δ^15^N)			
Source	DF	F Value	P > F	Source	DF	F Value	P > F
Genotype (G)	208	12.1	< 0.0001	Genotype (G)	208	3.1	< 0.0001
Environment (E)	1	834.3	< 0.0001	Environment (E)	1	2440.1	< 0.0001
G × E	194	1.6	< 0.0001	G × E	194	1.6	< 0.0001
Nitrogen Concentration [N]				Normalized DTR to AgNO_3_			
Source	DF	F Value	P > F	Source	DF	F Value	P > F
Genotype (G)	208	12.4	< 0.0001	Genotype (G)	210	1.5	< 0.0001
Environment (E)	1	284.0	< 0.0001				
G × E	194	1.7	< 0.0001

**Table 2:** Broad-sense heritability on an entry-mean basis for drought tolerance related traits evaluated

Trait	Both	GA-15	GA-16
	Heritability (%)		
Carbon Isotope Composition (δ^13^C)	62	61	72
Nitrogen Isotope Composition (δ^15^N)	17	24	40
Nitrogen Concentration [N]	64	63	73
Trait	Panel		
	Heritability (%)		
Normalized DTR to Silver Nitrate	17		

In general, these carbon and nitrogen related traits had fairly strong relationships with one another. Using best linear unbiased predictors (BLUP) values calculated from across both environments, correlations between the carbon and nitrogen related traits were from r = − 0.52 to 0.71 (Table 3). The most negative correlation (r = − 0.52) was between δ^13^C and δ^15^N, and the most positive correlation (r = 0.71) was observed between δ^13^C and N concentration (Table 3).

**Table 3:** Correlations among canopy wilting, carbon isotope composition (δ^13^C), nitrogen concentration, nitrogen isotope composition (δ^15^N), and normalized decrease in transpiration (NDTR) rate in response to silver nitrate (AgNO_3_)

	δ^13^C	δ^15^N	[N]	NDTR to AgNO_3_	Canopy Wilting^a^
δ^13^C	1.00^b^				
δ^15^N	−0.52	1.00			
[N]	0.71	−0.50	1.00		
NDTR to AgNO_3_	0.02	0.05	−0.02	1.00	
Canopy Wilting	− 0.08	− 0.02	0.08	0.00	1.00

^a^ Canopy wilting data are from [32]. These values were scored during the same field experiments as the present study

^b^ Best linear unbiased predictions (BLUPs) from across all replications and environments were used for the correlation calculations

PI 398823, a MG IV accession had the highest breeding value for δ^13^C using the sum across the two individual environments (Additional file 1). In addition, PI 416937, a slow-wilting check genotype, had a relatively high breeding value for this trait and ranked within the top 10% of genotypes tested (Additional file 1).

A MG VI accession from China, PI 567377B, had the most negative (favorable) breeding value for N concentration using the sum across both individual environments (Additional file 1). PI 471938, which was previously identified as a genotype possessing nitrogen fixation drought tolerance [21, 33], had the 40th lowest breeding value for N concentration (Additional file 1). Only 20 of the genotypes tested had negative breeding values for N concentration.

For δ^15^N, lower values would indicate that more nitrogen fixation from the atmosphere is occurring [26]. Forty-four of the genotypes evaluated in the panel had negative breeding values for δ^15^N, with PI 567386, a MG VI accession from China, having the most negative breeding value.

### Transpiration response to silver nitrate aquaporin inhibitor

**Fig. 2:**
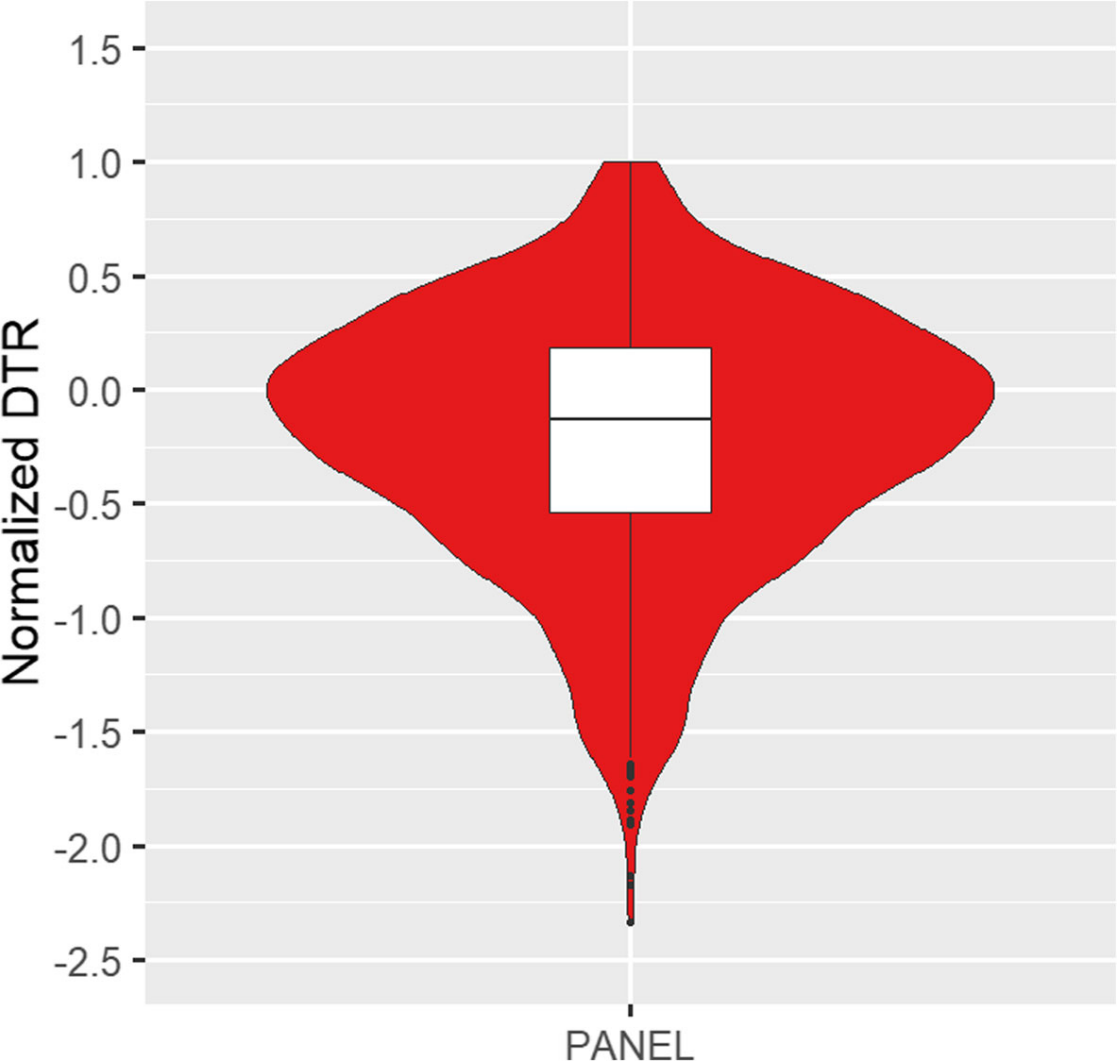
Violin plot with boxplot inside for normalized decrease in transpiration rate (NDTR) in response to silver nitrate treatment. Individual observations for the association panel across eight experimental replications are shown. DTR values were normalized by the highest DTR value in each separate experimental replication to calculate NDTR

Normalized decrease in transpiration rate (NDTR) values ranged from − 2.33 to 1.00 within individual replications (Fig. 2), and from − 0.99 to 0.48 based on genotype means. Genotype effects were statistically significant (*p* < 0.05) (Table 1), and broad-sense heritability on an entry-mean basis was 17% (Table 2). Using BLUP values across replications and environments, the relationships between NDTR in response to AgNO_3_ and the carbon and nitrogen related traits were also evaluated (Table 3). Silver nitrate NDTR was not correlated (r = − 0.02 to 0.05) with the previously described carbon and nitrogen related traits.

Twelve out of the 15 accessions with the most negative breeding values for transpiration response to AgNO_3_ originated from China (Additional file 1). PI 416937 was previously identified as a genotype with a transpiration response that is relatively insensitive to silver nitrate [30], and ranked 123rd based on NDTR breeding values.

### GWAS of carbon and nitrogen related traits

A total of 35 unique SNPs tagging 32 loci were identified either in individual environments or when using the BLUP calculated across both environments for δ^13^C (Additional file 2 and Table 4). Two SNPs for δ^13^C (ss715587736 and ss715587739) on Chr 4 were in the same genomic region, and were found in GA-15 and across both environments, respectively (Table 4). Of all other SNPs identified for δ^13^C, each SNP tagged a single genomic region, with the exception of two SNPs identified on Chr 4 and 16. The allelic effects across all significant (*p* < 0.0001; −log_10_*(P)* > 4) SNPs ranged from − 0.19 to 0.13 (Table 4), with all significant SNPs explaining a total of 29–44% of the variation, depending on the environment (Table 4).

**Table 4:** SNPs identified in a single environment or when using the BLUPs from both environments for carbon and nitrogen related traits that met the significance threshold level of -log_10_*(P)* > 4

Carbon Isotope Composition							
Locus^a^	Chr^b^	Pos^c^	SNP ID	-log_10_*(P)*	MAF^d^	Effect^e^	Env^f^
1	1	33,203,133	ss715578992	7.11	0.27	−0.12	Both
2	4	3,418,112	ss715587736	5.36	0.44	−0.09	GA-15
	4	3,425,900	ss715587739	6.90	0.46	−0.10	Both
3	4	46,166,265	ss715588297	4.40	0.45	0.06	GA-15
4	4	47,373,969	ss715588481	4.23	0.19	0.10	GA-15
	4	47,376,582	ss715588482	4.11	0.19	0.10	GA-15
5	5	37,563,155	ss715591464	5.44	0.42	−0.09	GA-16
6	6	6,576,054	ss715595435	5.61	0.47	0.08	Both
7	6	9,451,023	ss715595676	7.29	0.09	−0.18	GA-15
8	7	38,213,845	ss715597738	4.96	0.19	0.10	Both
9	8	19,267,914	ss715600198	6.65	0.19	−0.13	GA-15
10	8	19,518,756	ss715600277	5.87	0.32	0.11	GA-15
11	10	4,260,367	ss715607234	8.65	0.36	0.10	Both
12	10	21,586,075	ss715605850	5.49	0.47	0.10	GA-16
13	11	4,875,880	ss715610795	5.67	0.41	−0.11	GA-16
14	11	8,151,411	ss715611206	5.42	0.13	0.13	GA-16
15	12	458,748	ss715613097	5.79	0.45	−0.09	GA-16
16	12	38,049,740	ss715612828	6.25	0.21	−0.12	GA-16
17	13	28,776,094	ss715614695	4.89	0.36	−0.07	GA-15
18	14	12,079,082	ss715617567	4.45	0.47	−0.07	GA-15
19	14	47,854,709	ss715619453	5.15	0.43	−0.09	GA-15
20	15	40,841,088	ss715621829	4.25	0.12	−0.10	GA-15
21	15	47,257,859	ss715622121	6.99	0.07	−0.19	Both
22	15	47,349,730	ss715622149	7.93	0.12	−0.16	GA-15
23	16	3,557,974	ss715624794	4.71	0.33	0.08	Both
	16	3,566,872	ss715624799	5.15	0.37	0.09	Both
24	16	6,706,066	ss715625333	5.68	0.48	−0.08	Both
25	16	35,166,856	ss715624733	8.21	0.23	−0.12	Both
26	17	15,380,811	ss715626252	4.86	0.24	−0.12	GA-15
27	17	38,826,185	ss715627535	4.06	0.10	−0.12	GA-16
28	18	5,429,903	ss715631531	4.52	0.39	−0.08	GA-16
29	18	20,093,832	ss715629730	6.62	0.18	−0.12	GA-15
30	18	21,021,784	ss715629903	4.07	0.19	−0.12	GA-16
31	18	51,704,746	ss715631722	6.61	0.42	−0.09	Both
32	19	45,240,169	ss715635451	6.39	0.26	0.10	Both
Nitrogen Isotope Composition							
Locus	Chr	Pos	SNP ID	-log_10_*(P)*	MAF	Effect	Env
1	1	1,756,948	ss715578613	7.63	0.50	−0.05	Both
2	1	2,126,801	ss715578694	7.97	0.30	−0.10	GA-16
3	4	6,329,113	ss715589139	6.84	0.28	0.05	GA-15
4	6	21,606,676	ss715593886	5.99	0.12	0.07	GA-15
5	7	2,811,470	ss715597004	5.31	0.29	−0.05	Both
6	7	15,036,339	ss715596324	5.19	0.14	0.10	GA-16
7	9	3,732,795	ss715603834	8.26	0.22	−0.06	GA-15
8	9	45,017,460	ss715604529	7.87	0.32	−0.13	GA-16
9	10	2,699,011	ss715606028	5.10	0.08	−0.14	GA-16
10	10	7,565,702	ss715608519	6.18	0.20	−0.06	GA-15
11	13	7,212,966	ss715617100	5.54	0.07	0.08	GA-15
12	13	16,630,119	ss715616751	5.09	0.15	0.05	Both
13	14	30,072,552	ss715618124	9.22	0.11	0.11	Both
14	15	1,121,373	ss715620300	4.46	0.09	0.05	GA-15
15	15	13,304,091	ss715620571	4.71	0.23	−0.05	Both
16	15	49,446,994	ss715622476	6.61	0.33	0.04	GA-15
17	16	1,675,623	ss715623543	5.40	0.14	0.07	Both
18	17	11,003,712	ss715625747	4.24	0.34	−0.03	GA-15
19	18	8,504,254	ss715632791	4.11	0.20	−0.08	GA-16
20	19	39,924,653	ss715634905	7.68	0.49	−0.04	GA-15
21	19	45,292,930	ss715635458	4.44	0.37	0.08	GA-16
	19	45,292,930	ss715635458	5.35	0.37	0.06	Both
22	20	4,645,190	ss715638934	5.01	0.35	0.04	Both
23	20	39,218,472	ss715638011	4.19	0.24	−0.06	GA-16
Nitrogen Concentration							
Locus	Chr	Pos	SNP ID	-log_10_*(P)*	MAF	Effect	Env
1	1	51,706,358	ss715580153	5.08	0.09	−1.14	GA-16
2	2	1,482,658	ss715581317	4.16	0.24	0.62	GA-16
3	2	1,864,987	ss715581422	4.93	0.05	1.46	GA-16
4	3	2,475,142	ss715584877	5.60	0.45	−0.61	GA-15
5	3	2,945,818	ss715585023	8.03	0.15	−1.10	Both
6	3	37,336,737	ss715585803	8.25	0.27	−0.94	GA-15
7	6	16,421,594	ss715593518	9.09	0.28	−1.09	GA-15
8	6	16,706,510	ss715593613	4.49	0.27	−0.63	GA-15
9	7	4,232,510	ss715598067	7.40	0.15	1.16	GA-15
10	7	7,433,625	ss715598611	4.01	0.38	−0.56	GA-16
11	8	11,126,044	ss715599253	4.64	0.45	−0.58	GA-15
12	9	28,739,753	ss715603408	5.06	0.22	−0.79	GA-16
13	10	45,301,855	ss715607477	4.01	0.22	0.63	GA-15
14	11	34,311,552	ss715610522	4.50	0.33	0.58	GA-15
	11	34,311,552	ss715610522	4.14	0.33	0.53	Both
15	12	4,839,133	ss715613118	8.74	0.24	1.01	Both
16	12	8,677,962	ss715613605	5.53	0.10	1.05	GA-16
17	13	17,044,187	ss715616699	8.69	0.49	0.95	GA-16
	13	17,062,998	ss715616695	4.69	0.42	−0.55	Both
18	16	5,223,380	ss715625193	5.37	0.30	0.64	Both
19	16	8,026,492	ss715625562	5.89	0.17	−0.91	Both
20	16	32,772,447	ss715624545	7.06	0.46	0.99	GA-16
21	16	37,144,699	ss715624944	5.31	0.11	−1.17	GA-16
22	18	49,000,413	ss715631434	5.19	0.18	−0.84	GA-15
23	19	179,420	ss715635206	6.10	0.45	0.70	Both
24	19	48,043,481	ss715635757	4.57	0.38	0.68	Both
25	19	50,357,367	ss715636025	4.67	0.36	−0.66	GA-16
26	20	39,264,651	ss715638016	9.63	0.14	−1.33	GA-15

^a^ If multiple SNPs were identified in the same linkage disequilibrium (LD) block they were deemed part of the same locus (genomic region). Significant SNPs not part of the same LD block were deemed different loci controlling the trait

^b^ Chromosome

^c^ Glyma.Wm82.a2 physical position

^d^ Minor allele frequency

^e^ Allelic effects were calculated by taking the difference in mean phenotypic value between the two alleles at a particular SNP, and the direction, negative or positive, of the allelic effect estimates are relative to the alphabetical order of the nucleotides at each particular marker

^f^ Environment written as location-year

For δ^15^N, 23 loci were identified in the GWAS (Additional file 2 and Table 4). Depending on the environment, 36 to 51% of the phenotypic variation for δ^15^N was explained by the significant (p < 0.0001; −log_10_*(P)* > 4) SNPs. The allelic effects ranged from − 0.14 to 0.11 for the SNPs significantly associated with δ^15^N (Table 4). One SNP (ss715635458) was found for δ^15^N both in GA-16 and using the across both environments BLUPs (Table 4). All other SNPs identified tagged a single genomic region.

Twenty seven SNPs tagging 26 loci were identified in the GWAS for nitrogen concentration (Additional file 2 and Table 4). One SNP (ss715610522) was identified in both an individual environment (GA-15) and with the BLUP value from across both environments (Table 4). All other SNPs tagged a single genomic region, except for two SNPs (locus 17) on Chr 13. Allelic effects for nitrogen concentration ranged from − 1.33 to 1.46 (Table 4). Phenotypic variation explained (R^2^) across all significant SNPs for N concentration was 50, 35, and 21% for GA-15, GA-16, and across both environments (Both), respectively.

### GWAS for transpiration response to silver nitrate aquaporin inhibitor

**Fig. 3:**
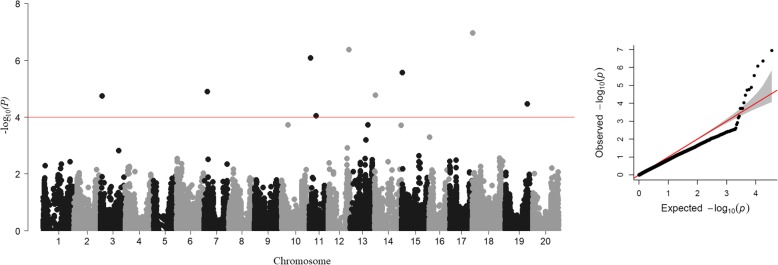
Genome-wide Manhattan and quantile-quantile plot for normalized decrease in transpiration rate (NDTR) in response to silver nitrate treatment. The X-axis is the genomic position of SNPs by chromosome across the soybean genome, and the Y-axis is the -log_10_ of the *p*-values obtained from the GWAS model. Significance threshold -log_10_*(P)* > 4 (red line). The quantile-quantile (QQ) plot to the right of the Manhattan plot shows the expected versus observed p-values of each SNP tested in the GWAS model

Nine SNPs tagging nine loci were significantly (*p* < 0.0001; −log_10_*(P)* > 4) associated with NDTR following silver nitrate treatment (Fig. 3 and Table 5). Thirty one percent of the phenotypic variation for the trait was explained by these nine SNPs. The allelic effects for these significant SNPs ranged from − 0.04 to 0.03 (Table 5).

**Table 5:** SNPs associated with normalized decrease in transpiration rate (NDTR) following silver nitrate treatment

Locus^a^	Chr^b^	Pos^c^	SNP ID	-log_10_*(P)*	MAF^d^	Effect^e^
1	3	2,996,563	ss715585043	4.73	0.17	0.03
2	7	5,960,839	ss715598416	4.89	0.13	0.03
3	11	2,124,435	ss715609637	6.08	0.46	−0.03
4	11	12,410,973	ss715609570	4.04	0.1	−0.03
5	12	38,552,678	ss715612877	6.37	0.47	−0.02
6	14	977,674	ss715620135	4.77	0.07	−0.04
7	15	1,906,120	ss715621180	5.56	0.33	−0.02
8	18	1,112,725	ss715628511	6.96	0.36	−0.03
9	19	41,078,499	ss715635080	4.46	0.41	0.02

^a^ If multiple SNPs were identified in the same linkage disequilibrium (LD) block they were deemed part of the same locus (genomic region). Significant SNPs not part of the same LD block were deemed different loci controlling the trait

^b^ Chromosome

^c^ Glyma.Wm82.a2 physical position

^d^ Minor allele frequency

^e^ Allelic effects were calculated by taking the difference in mean phenotypic value between the two alleles at a particular SNP, and the direction, negative or positive, of the allelic effect estimates are relative to the alphabetical order of the nucleotides at each particular marker

### Candidate genes for carbon and nitrogen related traits

For every trait evaluated, candidate genes were identified within plus or minus 10 kb (approximately spans the mean distance between all markers) of the SNPs with the lowest *p*-value (highest -log_10_*(P)*) in each environment and using across environments data. Eight, six, and seven candidate genes were identified for δ^13^C, δ^15^N, and N concentration, respectively, near these most significant SNPs (Additional file 3).

## Discussion

### Rationale for trait evaluation

In this study, a genetically diverse panel of over 200 soybean genotypes was evaluated for δ^13^C, δ^15^N, and nitrogen concentration from leaf samples collected in two different field environments. In addition, this panel was also evaluated for transpiration response to silver nitrate under high vapor pressure deficit conditions in a growth chamber. Using genome-wide association mapping, genomic regions were identified controlling each of these different drought tolerance related traits and the results were compared to previous mapping studies for these traits. In addition, genotypes in the panel were identified which possessed favorable breeding values for these drought tolerance related traits.

Carbon isotope composition can relate to photosynthetic tradeoffs that result from variation in water-saving capabilities. Nitrogen fixation can be highly sensitive to drought stress [14–16], and above-ground measurements such as nitrogen concentration and nitrogen isotope composition might relate to nitrogen fixation rate and soybean drought tolerance [17, 24, 25]. The amount of ^15^N found in a soybean plant would be decreased if it is actively fixing N_2_ from the atmosphere, and lower N concentrations have been shown to correlate with superior fixation during water deficits. However, given the high protein content of soybean, and the amount of nitrogen required to produce protein in seed, lower N concentrations could well be a poor trait for a soybean genotype to possess. Water-transporting proteins called aquaporins are involved in water movement through cell membranes [34], and populations of aquaporins in soybean lines can vary as detected by transpiration response to chemical inhibitors such as silver nitrate [30, 31, 35]. It is hypothesized that insensitivity to silver nitrate is correlated with the limited leaf hydraulic conductance trait, a beneficial trait associated with water conversion and improved drought tolerance in certain environments [28, 29]. All of these traits were evaluated in the current study in order to develop insight about the genetic architecture of these drought tolerance related traits and identify germplasm with favorable breeding values for these traits.

### δ^13^C, δ^15^N, and N concentration

Values for δ^13^C were in a similar range to those observed in two previous carbon isotope association mapping studies [11, 12] (Fig. 1). The range of values observed for nitrogen concentration was wider and concentrations were higher compared to those observed in a previous study [26]. Direct comparisons to [26] were not able to be made for δ^15^N due to differences in the units used for these measurements. Analyses of variance (ANOVA) showed that genotype, environment, and their interaction were statistically significant (*p* < 0.05) for all carbon and nitrogen related traits evaluated with the association panel (Table 1). Although these genotype-by-environment interactions were significant (*p* < 0.05), correlations were generally high between the two environments. Correlations for δ^13^C and nitrogen concentration were all above r = 0.70 between the two environments tested, indicating the genotypes performed similarly across environments. The lowest correlation was for δ^15^N at r = 0.28, which suggests this trait could be subject to environmental influence, such as nitrogen levels in the soil.

Heritability for δ^15^N was substantially lower and ranged from 17 to 40% (Table 2). This lower heritability for δ^15^N could potentially be explained by the fact that we did not adjust our values to a non-nodulating reference crop, and that these values are also affected by field variation in soil nitrogen concentration [36]. However, heritability estimates for all of these carbon and nitrogen related traits are comparable to the values observed in other studies [11, 12, 26].

### Transpiration response to AgNO_3_

Low or negative DTR to silver nitrate values (transpiration less affected by AgNO_3_) have been previously correlated with limited leaf hydraulic conductance, which is a beneficial trait in certain drought stress environments [29]. Given the hypothesis that silver nitrate blocks only specific aquaporins and reduces transpiration, and that most previously reported DTR values were positive, we observed an unexpected distribution of NDTR values given that many of the genotypes we tested had negative non-normalized DTR (negative NDTR). This could indicate that silver nitrate blocked some aquaporins as expected, but in some genotypes this blockage resulted in a stimulus in the number or activity of other silver-insensitive aquaporins. However, this hypothesis needs further experimental investigation.

Analyses of variance found that genotype effects were statistically significant (*p* < 0.05) (Table 1), and heritability for this trait was 17% (Table 2). This low heritability estimate could have been a result of a technical issue or that this phenotyping method may not be a reliable proxy for limited leaf hydraulic conductance, and would make it difficult for soybean breeders to make effective selection for this trait. One potential technical issue which could have explained the low heritability observed was variation in VPD throughout each experimental replication and between each of the eight replications, as well as VPD values lower than the desired 3.00 kPa for our protocol. As shown in Table 6, average VPD by replication ranged from 1.56 to 2.33 kPa. In addition, VPD variation within each replication was relatively stable, but varied by as much as 0.5 kPa during a single replication due to the size of the walk-in growth chamber and its ability to maintain the environmental settings we aimed to achieve in the protocol. While temperature remained relatively constant throughout the experiments, relative humidity (RH) was more variable, and was the primary driver in the varying VPD observed (Table 6). Given the genotypes tested were a diverse panel from different maturity groups and geographic origins, there was some variation in the size of the plants as they were growing in the greenhouse in preparation for the experiments. This variation in size was accounted for in our DTR calculations, because each plant’s difference in transpiration rate between water and silver nitrate solution was relative to itself. However, it is still worth noting that plant size differences could cause some degree of soil moisture deficit in the relatively small pots we used to grow the plants to V3-V4 stage, and may be another factor to explain the low heritability we observed. In addition, during the process of cutting the shoot of the soybean plants from the roots it is possible that some plants were embolized. However, as part of our protocol, we made a second cut underwater away from our initial cut to help potentially avoid this issue.

**Table 6:** Summary of transpiration response to silver nitrate treatment experiments for the association panel

Replicate	Measurement Date	Average VPD^a^	Average Temperature^b^	Average RH^c^
1	4/1/2015	2.33	30.37	46.23
2	4/10/2015	1.92	30.33	55.55
3	4/15/2015	1.91	30.31	55.78
4	4/22/2015	2.24	30.27	48.16
5	6/23/2015	1.63	30.51	62.61
6	6/24/2015	1.56	30.48	64.23
7	10/20/2015	2.29	30.28	45.40
8	3/31/2016	1.74	29.36	57.71

Environmental parameters were measured with two data loggers from the time of the first weighing of the de-rooted shoots in deionized water to the final weighing of the de-rooted shoots in silver nitrate solution. Values in table are average of the two data logger measurements

^a^ Vapor pressure deficit (VPD) in kPa

^b^ Temperature in degrees Celsius

^c^ Relatively humidity (RH) percentage

### Comparison to previous mapping results for carbon and nitrogen traits

Given that FarmCPU uses the most significant markers as covariates in the GWAS model, SNPs are seldom identified within the same LD block for an environment-specific dataset. However, two genomic regions were found both in individual environments and when using the across both environments BLUP data for these carbon and nitrogen related traits. Significant (*p* < 0.0001; −log_10_*(P)* > 4) SNPs for carbon and nitrogen related traits were found on all 20 soybean chromosomes (Table 4).

**Fig. 4:**
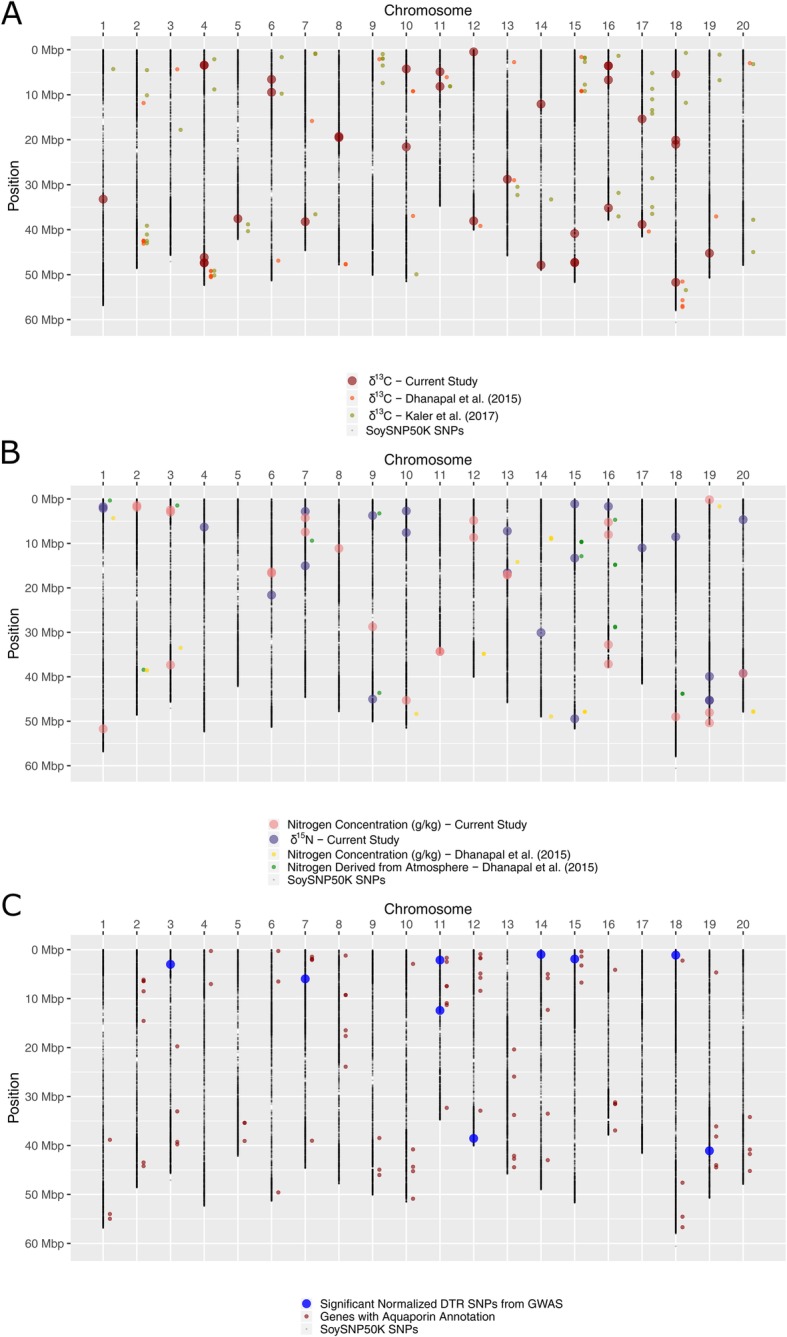
Location and comparison of SNPs significantly associated with drought tolerance related traits. Physical positions are based on the Glyma.Wm82.a2 version of the soybean genome. SNPs identified in GWAS from current study that met -log_10_*(P)* > 4 significance threshold are shown as larger circles for **a**) carbon isotope composition (δ^13^C), **b**) Nitrogen concentration and nitrogen isotope composition (δ^15^N), and **c**) normalized decrease in transpiration rate (NDTR) in response to silver nitrate treatment. Smaller circles represent SNPs identified in **a**) [11, 12], **b**) [26] that were converted from version 1 to 2 physical positions of the soybean genome assembly, and **c**) location of gene models with the term “aquaporin” in their functional annotation from Phytozome v12.1. BARC_1.01_Gm20_46575262_G_A identified for nitrogen concentration in [26] does not have a perfect match in the version 2 assembly, and therefore was excluded from this comparison

Previously identified QTLs for CID are numbered with their approximate physical positions on the SoyBase website (www.soybase.org). Locus 32 identified with GWAS for δ^13^C in the current study is found within the CID 1–5 QTL on Chr 19 identified in [13] (Table 4). A comparison of SNPs significantly associated with δ^13^C from two previous association mapping studies [11, 12] and the current study was conducted (Fig. 4a). Two SNPs on Chr 6 and 11 from the current study are near significant markers identified in [12], and one SNP on Chr 13 and another SNP on 18 were found near the significant SNPs for δ^13^C in [11].

No QTLs for δ^15^N identified with linkage mapping are reported on the SoyBase website. One previous linkage mapping study for foliar nitrogen concentration identified four QTLs, of which one QTL on Chr 16 was 256 kb away from locus 21 identified in the current study [22]. A comparison of SNPs identified for nitrogen related traits in a previous association mapping study [11] and the current study was also performed (Fig. 4b). SNPs on Chr 9 and 15 were found in common for δ^15^N in the current study and nitrogen derived from the atmosphere (Ndfa) in [26]. No SNPs were within 1 Mb of previously identified genomic regions for nitrogen concentration. Additionally, when making comparisons only across studies and different nitrogen related traits, only two regions on Chr 15 and 16 had common SNPs within 1 Mb of each other. Within the current study only, two regions contained nitrogen related significant (*p* < 0.0001; −log_10_*(P)* > 4) SNPs within 1 Mb of each other on Chr 13 and 20 (Table 4). The relatively small number of consistent associations across these studies could be due to differences in the maturity groups tested or the tissue collection method. However, the consistent QTLs and genomic regions across environments, studies, and traits, along with SNPs explaining a high amount of phenotypic variation in the current study could be useful as breeding targets for these carbon and nitrogen drought tolerance related traits.

### Genetic mapping for transpiration response to AgNO_3_ and proximity of identified regions to aquaporin gene models

This is the first report of association mapping for this trait to the authors’ knowledge in any crop species. A previous QTL mapping study for limited leaf-hydraulic-conductance identified QTLs on Chr 3, 5, 10, and 12 [31]. The locus identified on Chr 12 in the current study is located approximately 2 Mb away from the Chr 12 QTL from that previous study. A lack of overlap in the genomic regions observed in these two studies could be due to differences in the populations utilized for the mapping, and could also be affected by the low heritability for this trait (Table 2). A search on Phytozome for gene models with a functional annotation which contained the word “aquaporin” was also conducted given the hypothesized relationship between this limited leaf hydraulic conductance trait and aquaporins, and found 88 gene models. The physical locations of these gene models and the loci identified in the current study with association mapping were compared (Fig. 4c). Three SNPs identified in the GWAS were within 1 Mb of four gene models with an aquaporin functional annotation. These regions could be further investigated to see how this trait relates to aquaporins.

### Candidate genes at identified genomic regions for carbon and nitrogen related traits

A total of 21 gene models were identified near the most significant SNP across each trait and environment tested. A gene model located at locus 11 for carbon isotope composition, Glyma.10 g047500, is a protein phosphatase 2C family protein (Additional file 3). This gene family has been shown to function at the intersection of drought, oxidative, and heat shock stresses in tobacco [37]. The gene model Glyma.09 g043900 is a transducing/WD40 repeat-like superfamily protein located near locus 9 (ss715603834) associated with nitrogen isotope composition (Additional file 3). A report in *Arabidopsis thaliana* showed that a member of the WD40 gene family functions in drought stress tolerance by modulating nitric oxide accumulation and stomatal closure [38]. A C2H2-type zinc finger family protein gene (Glyma.12 g065800) located at locus 15 is associated with nitrogen content (Additional file 3). In rice, a zinc finger transcription factor, drought and salt tolerance (DST), was shown to play a role in stomata-regulated abiotic stress tolerance [39]. These gene models could be potential targets for understanding and improving these drought tolerance related traits given their relationship with drought stress tolerance response or enhancement.

### Relationship between drought tolerance related traits

Another measurement related to soybean drought tolerance, canopy wilting, was added to the Table 3 correlation matrix using data from [32]. This additional data from the same field experiments provides another trait to compare to carbon and nitrogen related traits and NDTR in response to silver nitrate treatment. Canopy wilting and NDTR to silver nitrate had relatively low correlations with each of the other traits evaluated and with one another. A previous study also found that there was not a consistent relationship among genotypes within slow- or fast-canopy wilting groups and CID [40]. Drought tolerance is a complex, quantitative trait, so it is expected that multiple different traits and loci are responsible for soybeans’ ability to withstand water deficit stress.

### Breeding implications

Many different genotypes were identified in the current study with favorable breeding values for drought tolerance related traits and could be utilized by breeders to improve soybean drought tolerance directly with forward breeding or be used as parents to create mapping populations to further understand the genetic architecture for these traits. Genotypes with positive breeding values for δ^13^C, negative breeding values for N concentration, negative breeding values for δ^15^N, and accessions with lower NDTR values and low negative breeding values could be candidate parents to use for drought-tolerance improvement in a soybean breeding program. However, the challenge as a breeder would be to determine which trait(s) to target given the quantitative nature of the genetic architecture for many traits that could lead to soybean drought tolerance improvement, and some of these traits could be associated with poor agronomic performance.

In addition, accessions in the current study often had favorable breeding values for certain traits, but then also had less favorable breeding values for other traits (Additional file 1). As a reference point, PI 416937, a genotype previously identified as possessing the slow canopy-wilting trait [41], was ranked as the 133rd best accession tested based on an overall median rank across breeding value ranks for canopy wilting, carbon isotope composition, nitrogen concentration, nitrogen isotope composition, and NDTR in response to silver nitrate (Additional file 1). It ranked 69th best for canopy wilting and 15th best for carbon isotope composition, but ranked 189th for nitrogen concentration, 140th for nitrogen isotope composition, and 123rd best for transpiration response to silver nitrate (Additional file 1). One hundred thirty-two accessions with overall median ranks lower than PI 416937 were identified in this research (Additional file 1). To make selections based on multiple traits an index accounting for trait heritability, economic importance, and genetic and phenotypic correlations among the traits would likely need to be employed with consideration for phenotyping costs and genotype by environment interactions for these traits. Ultimately, a breeder may need to weight traits according to which would provide the best drought tolerance in their given target environment, and then utilize the germplasm and genomic regions identified for that specific trait.

## Conclusions

Genome-wide association analyses were conducted for δ^13^C, δ^15^N, and nitrogen concentration from two environments using over 200 genetically diverse soybean genotypes. Thirty two, 23, and 26 loci were identified for δ^13^C, δ^15^N, and nitrogen concentration, respectively. One locus detected with the GWAS for δ^13^C was co-located with a previously identified QTL for CID, and four SNPs were near SNPs found in previous association mapping studies. Two SNPs for δ^15^N were found in the GWAS near genomic regions identified in an association mapping study for nitrogen related traits. Nine SNPs tagging nine loci were identified with a GWAS approach for normalized DTR to silver nitrate, and three of the SNPs identified were found near four aquaporin related gene models. Breeding values calculated with the significant SNPs from the GWAS enabled the identification of accessions which possess favorable combinations of alleles for these drought tolerance related traits. The genomic regions and germplasm identified in this study, especially those found in common across environments, studies, and traits, can be used to understand the genetic architecture for these traits and by soybean breeders to improve drought tolerance.

## Methods

### Soybean populations

An association panel of 211 genetically diverse soybean genotypes was evaluated for transpiration response to a silver nitrate solution. The panel was previously described in [32], but with the addition of two lines and replacement of 10 other lines that did not produce enough seed for the field evaluations of drought tolerance related traits described in [32] and also in the current study. This panel was selected based on SoySNP50K genotype data to be genetically diverse, consisted mostly of maturity group (MG) VI-VIII plant introductions, and included drought tolerant and susceptible genotypes. One hundred ninety-five and 205 of the soybean genotypes described in [32] were evaluated in 2015 and 2016 in Athens, GA, respectively, for carbon and nitrogen related traits in the field. The majority of these lines had not previously been evaluated for drought-tolerance related traits, and are later maturing lines than those previously tested (MG IV) and used for association mapping of these traits [11, 12, 26].

### Isotope analysis and sample collection

Leaf samples were collected from field plots of the association panel grown in Athens, GA in 2015 (GA-15) and 2016 (GA-16) and used for stable isotope analysis. More information about sowing dates, row spacing, and management of these plots can be found in [32]. Based on soil sample testing, no fertilizer was added to the field in 2015, and a 4–15-30 fertilizer was applied at a rate of 392 kg ha^− 1^ in 2016 prior to sowing. These plots were grown under rain-fed conditions and experienced intermittent drought stress periods in both years. In 2015, the leaf samples were collected on 23 September and on 12 September in 2016. All of the soybean genotypes in the panel were in reproductive growth stages (R3-R6) at the time of sample collection. Five leaves were randomly selected from each of the two-row plots at the third trifoliolate leaf below the top of the plants. These leaves were placed in seed envelopes, and stored in a − 20^0^ C freezer until they could be processed at a later date. For isotope analysis, 100–150 samples were processed at one time by transferring leaf samples to 50 ml Falcon tubes and placing them in a lyophilizer for two days to freeze dry. The samples were then ground to a fine powder by placing 4.5 mm zinc plated BBs in the tubes and grinding them using a Geno/Grinder (SPEX SamplePrep, Metuchen, New Jersey, USA). Immediately before using this ground leaf tissue for isotope analysis, the tubes were placed in a drying oven to ensure all residual moisture was removed. In an effort to further keep out moisture, the Falcon tube caps were wrapped with Parafilm immediately after this second drying step.

Stable isotope analysis was then performed using a Carlo Erba NA1500 CHN combustion analyzer coupled to a Delta V isotope ratio mass spectrometer via the Conflo III open split interface. Three experimental replications of the dry leaf tissue of each genotype were analyzed at the Center for Applied Isotope Studies, University of Georgia, Athens, GA. A detailed protocol for the procedure can be found at http://sisbl.uga.edu/ratio.html. The quantity of ^13^C in the leaf samples was compared to a reference standard Pee Dee Belemnite, and these δ^13^C values were used for further analyses. δ^13^C was expressed in units per mil (‰) using the following equations [4]:

$$ R={}^{13}{\mathrm{CO}}_2/{}^{12}{\mathrm{CO}}_2 $$

$$ {\updelta}^{13}\mathrm{C}\ \left({\mbox{\fontencoding{U}\fontfamily{wasy}\selectfont\char104}} \right)=1000\ \left({R}_{\mathrm{sample}}-{R}_{\mathrm{standard}}\right)/{R}_{\mathrm{standard}} $$

The quantity of ^15^N in the leaf samples was compared to air and expressed in units per mil (‰) according to the following equations:

$$ R={}^{15}\mathrm{N}/{}^{14}\mathrm{N} $$

$$ {\updelta}^{15}\mathrm{N}\ \left({\mbox{\fontencoding{U}\fontfamily{wasy}\selectfont\char104}} \right)=1000\ \left({R}_{\mathrm{sample}}-{R}_{\mathrm{air}\ \mathrm{N}2}\right)/{R}_{\mathrm{air}\ \mathrm{N}2} $$

Nitrogen concentration was expressed as g kg^− 1^.

### Evaluation of response to silver nitrate inhibitor

Soybean plants for evaluation of transpiration response to silver nitrate were grown in a greenhouse at the University of Georgia in Athens, GA, USA under a 16 h day and eight hours night lighting regime. Three seeds of each genotype were sown in 32 oz. styrofoam cups using a Fafard 2B soil media (Sun Gro Horticulture, Agawam, MA, USA). Approximately 1.5 weeks after seedling emergence, the plants were thinned to one plant per cup and maintained under well-watered conditions by watering each pot twice daily until the soil reached water holding capacity. Once the soybean plants reached the V3-V4 growth stage (approximately four weeks after sowing), the tests for response to the silver nitrate inhibitor began [29].

The tests were conducted over two days. In the afternoon of the first day, the soybean plants were removed from their growing media in the greenhouse and de-rooted using clippers. A second cut on the stem was then made underwater adjacent (1–3 cm away) to the first cut using a razor blade. The remaining shoot was then placed in a 250 mL Erlenmeyer flask filled with deionized water and the mouth of the flask was sealed with Parafilm to avoid water evaporation. Plants in flasks were then placed in a walk-in Conviron growth chamber at approximately 20^0^ C and 60% relative humidity (RH) overnight in dark conditions.

In the morning of day 2, the growth chamber settings were adjusted to turn the lights on, raise temperature to 30^0^ C, and decrease RH to 30% to obtain a higher vapor pressure deficit (VPD) in the growth chamber. The observed VPD for the chamber was between 1.56–2.33 kPa across replications of the experiment (Table 6). The plants were allowed to acclimate to the higher VPD condition for 60 min. Then, each flask/soybean was weighed inside the growth chamber using a balance with a resolution of 0.001 g in order by flask number. Sixty min after the first weighing, they were weighed again in the same order to determine the transpiration rate in water (TR_W_). Each soybean shoot was then transferred to a 60 mL amber glass bottle containing a 200 μM solution of silver nitrate (AgNO_3_) under semi-dark conditions. This AgNO_3_ solution concentration was previously shown to best differentiate the transpiration response of drought tolerant versus susceptible soybean plants in [29]. Parafilm was again used to seal the mouth of the amber bottles to avoid evaporation and spilling of any chemical. Then, the plants were returned to the growth chamber and allowed to acclimate to the inhibitor treatment for 60 min. The amber bottles with shoots were then weighed for their initial weight in order by bottle number. After approximately 120–160 min, the bottles were reweighed in bottle order to determine the transpiration response to the silver nitrate inhibitor (TR_I_). Differences in the amount of time that elapsed between weight measurements were accounted for in the TR_W_ and TR_I_ calculations by changing the denominator in increments of minutes. Decrease in transpiration rate (DTR, %) was then calculated as follows:

$$ \mathrm{DTR}=100\times \frac{\left({\mathrm{TR}}_{\mathrm{W}}-{\mathrm{TR}}_{\mathrm{I}}\right)}{{\mathrm{TR}}_{\mathrm{W}}} $$

Due to limitations in the size of the walk-in growth chamber and ability to weigh the flasks/bottles in an orderly and timely fashion, eight separate replications of this experiment were conducted (Table 6). Each replication consisted of the entire panel of 211 soybean genotypes, and the flask/bottle order was randomized for each replication. To account for small differences in the range of DTR among the eight replicate experiments due to plant size and environmental differences with each replication, the results were normalized against the genotype with the highest DTR value within each replication using the following equation:

$$ \mathrm{Normalized}\ \mathrm{DTR}\ \left(\mathrm{NDTR}\right)\ \mathrm{within}\ \mathrm{Each}\ \mathrm{Replication}={\mathrm{DTR}}_{\mathrm{Genotype}}/{\mathrm{DTR}}_{\mathrm{Genotype}\ \mathrm{with}\ \mathrm{Highest}\ \mathrm{DTR}} $$

### Genotype data and quality control

The association panel was genotyped with the SoySNP50K iSelect BeadChip [42]. DNA extraction and genotyping procedures for this panel were conducted as described in [32]. A total of 42,079 genome-wide SNP markers resulted from the genotyping effort, with most marker data being downloaded from SoyBase [43]. Markers with minor allele frequencies (MAF) lower than 0.05 were eliminated leaving 35,262 SNP markers for the association analysis of transpiration response to silver nitrate. For the carbon and nitrogen related traits, 35,234 (Both), 35,101 (GA-15), and 35,219 (GA-16) markers were used after eliminating markers with MAF lower than 0.05. The number of markers varied, because certain SNPs with a MAF close to 0.05 were either included or excluded depending on the number of entries tested in the given environment. Physical positions are based on the Glyma.Wm82.a2 version of the soybean genome.

### Statistical analyses

Analyses of variance (ANOVA) was conducted using PROC GLM in SAS version 9.4 (SAS Institute Inc., Cary, NC, USA). For the response variables relating to carbon and nitrogen traits, genotype was treated as a fixed effect, and environment, genotype-by-environment interaction, and replication within environment were random effects. For transpiration response to silver nitrate, a model was created with genotype as a fixed effect and replication as a random effect, with NDTR as the response variable. Broad-sense heritability was calculated on an entry-mean basis according to [44] with the variance components being calculated with PROC MIXED of SAS 9.4 using a model where all variables were treated as random.

Best linear unbiased predictors (BLUPs) were calculated from both across and within environments and used as the phenotype values for subsequent GWAS analyses. The BLUP calculations for carbon and nitrogen related traits across both environments were performed using JMP Pro (JMP®, Version 13, SAS Institute Inc., Cary, NC, USA). The model was built by treating genotype, environment, genotype-by-environment, and replication within environment as random variables using the Standard Least Squares personality and REML method. For individual environments for carbon and nitrogen related traits and transpiration response to silver nitrate, genotype and replication were used as variables and treated as random to calculate BLUPs.

### Genome-wide association analyses

Fixed and random model Circulating Probability Unification (FarmCPU) was used to perform the genome-wide association analyses for all traits evaluated [45]. FarmCPU is an R package that implements a multiple loci linear mixed model incorporating a modified mixed linear model that includes the most significant markers as covariates. It uses fixed and random effect models iteratively to help reduce potential confounding between the markers and kinship. This model has previously been successfully utilized in soybean genome-wide association analyses to identify genomic regions controlling canopy wilting [32, 46], carbon and oxygen isotope ratios [12], and resistance to *Sclerotinia sclerotiorum* [47].

Manhattan plots were visualized with the ‘qqman’ [48] and ‘CMplot’ R packages using the *p*-values generated from the FarmCPU output. The significance threshold (*p* < 0.0001; −log_10_*(P)* > 4) was used to determine if SNPs were significantly associated with the traits of interest. This threshold is less stringent than a Bonferroni-corrected threshold, but is more stringent than many other soybean GWAS studies using 50 K SNP genotyping data [12, 46, 49, 50]. It is also near the point at which the p-values deviated from the linear expected p-values in the quantile-quantile (QQ) plots (Additional file 2). Days to flowering (DTF) was recorded in both field environments as the number of days from sowing until 50% of the plants in a plot reached the first bloom (R1) growth stage. The carbon and nitrogen related traits evaluated had relatively strong correlations (data not shown) with DTF in both environments, so DTF was used as a fixed effect covariate, along with the first four genetic principal coordinates, in the GWAS to account for this correlation and population structure, respectively.

Haploview version 4.2 software [51] was used to calculate pairwise estimates of D′ and r^2^ and estimate linkage disequilibrium (LD) blocks. Using D′ > 0.8 to extend the spine, LD blocks were identified by chromosome with the Solid Spine of LD option. These LD blocks were used to determine if significant (*p* < 0.0001; −log_10_*(P)* > 4) SNPs that are physically close (less than 1 Mb) were at the same locus (genomic region) controlling the trait of interest. Significant SNPs not part of the same LD block were deemed different loci controlling the trait. Allelic effects were calculated by taking the mean difference in phenotypic values for the trait between the two alleles at a particular SNP, and were provided as part of the FarmCPU output. A negative effect value indicates that an individual possessing the second nucleotide alphabetically for this SNP would have lower phenotypic values, whereas a positive effect value would have higher phenotypic values. The direction, negative or positive, of the effect is based on how the genotype data was converted from HapMap to numerical format using GAPIT [52] prior to conducting the GWAS with the numerically formatted genotype data in FarmCPU. Since BLUP values were used as the phenotype in the GWAS, the allelic effects reported are based on these BLUP values rather than the original raw data. Phenotypic variation explained (R^2^) by significant (*p* < 0.0001; −log_10_*(P)* > 4) SNPs was calculated using a linear regression in R. The model lm(BLUP ~ SNP_1_ + SNP_2_ + …) was used to determine the total amount of phenotypic variation explained by all significant SNPs for a given trait in a particular environment.

Breeding values for the traits were calculated by summing the allelic effects for all significant (*p* < 0.0001; −log_10_*(P)* > 4) SNPs in each individual environment and with the across environments BLUPs. Breeding values across the individual environments were also summed and used for comparisons. Allelic effects for a given SNP were considered negative if the allele contributed to lower phenotypic values, and positive if it increased phenotypic values. Heterozygous and missing allele calls were not included in the breeding value calculation.

### Identification of gene models at significant SNPs and with aquaporin functional annotation

Using SoyBase [43], candidate genes along with their functional annotation and gene ontologies were identified near the most significant (*p* < 0.0001; −log_10_*(P)* > 4) SNPs from GWAS in each environment and across environments for each of the carbon and nitrogen related traits. Glyma2.1 gene models within plus or minus 10 kb of the SNP physical position were recorded and further investigated. The median distance between SNP markers used in the GWAS was 9 kb, and the mean distance was 26 kb. Although identifying all gene models in LD with significant SNPs would be ideal, the efforts were focused on models in close proximity (within plus or minus 10 kb), which approximately spans this distance between markers.

Given the hypothesized relationship between transpiration response to silver nitrate and sensitivity of aquaporin populations in soybean [29, 30, 53], a search for the term “aquaporin” was performed in Phytozome v12.1 for the *Glycine max* Wm82.a2.v1 version of the soybean genome. This identified 88 gene models which had “aquaporin” in their functional annotation. In comparison, 82 of these gene models were also found when searching for “aquaporin” on the SoyBase website (www.soybase.org). The physical locations of the full list of 88 gene models having an aquaporin annotation from Phytozome were used to make comparisons between the significant (*p* < 0.0001; −log_10_*(P)* > 4) SNPs identified for transpiration response to silver nitrate from the GWAS results to see if any aquaporin genes were in or near these regions.

## Supplementary information

**Additional file 1.** Breeding value ranks for accessions tested for canopy wilting, carbon isotope composition (δ^13^C), nitrogen concentration, nitrogen isotope composition (δ^15^N), and normalized decrease in transpiration rate (NDTR) in response to silver nitrate (AgNO_3_) treatment.

**Additional file 2.** Genome-wide Manhattan plots for carbon and nitrogen related traits.

**Additional file 3.** Candidate genes and their functional annotation identified using the Glyma2.1 gene models in SoyBase within plus or minus 10 kb of SNPs significantly associated with carbon and nitrogen related traits.

## Data Availability

SNP marker genotypes for accessions included in the association panel can be retrieved from SoyBase (www.soybase.org). All other datasets generated and/or analyzed during the current study are not publicly available, but are available from the corresponding author on reasonable request.

## Abbreviations

ANOVA: Analyses of variance
BLUP: Best linear unbiased predictors
CID: Carbon isotope discrimination
DTF: Days to flowering
DTR: Decrease in transpiration rate
GWAS: Genome-wide association studies
LD: Linkage disequilibrium
MAF: Minor allele frequency
MG: Maturity group
NDTR: Normalized decrease in transpiration rate
QQ: Quantile-quantile
QTL: Quantitative trait locus
RH: Relative humidity
SNP: Single nucleotide polymorphism
VPD: Vapor pressure deficit
