# Supplementary material for: Unraveling the genetic architecture for carbon and nitrogen related traits and leaf hydraulic conductance in soybean using genome-wide association analyses
Source: BMC Genomics. 2019 Nov 6;20:811. doi: 10.1186/s12864-019-6170-7 (PMC6836393; doi:10.1186/s12864-019-6170-7)
Supplement: Supplementary file 1 — Additional file 1. Breeding value ranks for accessions tested for canopy wilting, carbon isotope composition (δ13C), nitrogen concentration, nitrogen isotope composition (δ15N), and normalized decrease in transpiration rate (NDTR) in response to silver nitrate (AgNO3) treatment. [file 12864_2019_6170_MOESM1_ESM.docx]

**Additional file 1**

Breeding value ranks for accessions tested for canopy wilting, carbon isotope composition (δ^13^C), nitrogen concentration, nitrogen isotope composition (δ^15^N), and normalized decrease in transpiration rate (NDTR) in response to silver nitrate (AgNO_3_) treatment. Only genotypes that were tested for all five traits are shown in this table.

| Accession | Name | Country | MG | Canopy Wilting | δ^13^C | [N] | δ^15^N | NDTR to AgNO_3_ | Median Rank |
| --- | --- | --- | --- | --- | --- | --- | --- | --- | --- |
|  |  |  |  | ----Breeding Value Rank^a^---- | | | | |  |
| PI398823 | - | South Korea | IV | 24 | 1 | 5 | 110 | 142 | 24 |
| PI567386 | Huang da dou (1) | China | VI | 185 | 182 | 26 | 2 | 25 | 26 |
| PI341248 | Sangalo | Tanzania | IX | 29 | 29 | 84 | 18 | 64 | 29 |
| PI567036 | - | Morocco | IX | 26 | 4 | 175 | 36 | 202 | 36 |
| PI203406 | - | South Africa | VIII | 123 | 9 | 38 | 106 | 13 | 38 |
| PI341246 | CNS | Tanzania | IX | 178 | 21 | 144 | 10 | 39 | 39 |
| PI567316B | (Hong huang dou) | China | VI | 38 | 40 | 109 | 29 | 83 | 40 |
| PI548983 | Tracy | United States | VI | 10 | 20 | 140 | 42 | 194 | 42 |
| PI374220 | Geduld | South Africa | VI | 176 | 35 | 138 | 44 | 31 | 44 |
| PI567326B | (Huang dou) | China | VI | 45 | 154 | 67 | 4 | 13 | 45 |
| PI603588 | Jing si dou | China | V | 61 | 127 | 48 | 15 | 13 | 48 |
| PI219698 | Kulat | Pakistan | VI | 72 | 89 | 49 | 23 | 43 | 49 |
| PI548980 | Hood | United States | VI | 4 | 141 | 41 | 84 | 50 | 50 |
| PI471938 | - | Nepal | V | 33 | 141 | 40 | 141 | 50 | 50 |
| PI322694 | Hernnon | Zimbabwe | VI | 51 | 61 | 18 | 30 | 174 | 51 |
| PI341244B | (Yellow Kedele) | Tanzania | IX | 39 | 106 | 69 | 53 | 32 | 53 |
| PI423927 | Tousan 93 | Japan | IV | 54 | 51 | 36 | 71 | 145 | 54 |
| PI159093 | 34S51 | South Africa | VII | 177 | 54 | 29 | 46 | 131 | 54 |
| PI434980B | (Indo 180) | Central African Republic | IX | 124 | 8 | 124 | 27 | 55 | 55 |
| PI567334 | Jiang dou zi | China | VI | 113 | 36 | 56 | 96 | 6 | 56 |
| PI567406B | (Wu se da dou) | China | VI | 36 | 56 | 191 | 123 | 22 | 56 |
| PI381663 | Kakira 1 | Uganda | VI | 201 | 13 | 163 | 31 | 56 | 56 |
| PI330633 | - | South Africa | VII | 175 | 11 | 206 | 57 | 39 | 57 |
| PI578495 | Jin dou No. 4 | China | IV | 37 | 58 | 33 | 142 | 208 | 58 |
| PI567356 | Zao bai huang dou | China | VI | 5 | 27 | 181 | 59 | 61 | 59 |
| PI322689 | Improved | Angola | VII | 181 | 53 | 105 | 62 | 60 | 62 |
| PI486328 | Birsa Soybean-1 | India | VIII | 41 | 62 | 122 | 192 | 5 | 62 |
| PI407738 | - | China | VI | 125 | 198 | 23 | 63 | 33 | 63 |
| PI603512 | Jin man dou | China | VI | 64 | 125 | 22 | 160 | 28 | 64 |
| PI374180 | - | India | VIII | 49 | 119 | 55 | 75 | 65 | 65 |
| PI429329 | - | Nigeria | VII | 139 | 110 | 65 | 20 | 53 | 65 |
| PI360846 | Shiroge-9 | Japan | IV | 66 | 82 | 58 | 56 | 102 | 66 |
| PI430737 | Oribi | Zimbabwe | VII | 203 | 57 | 37 | 72 | 66 | 66 |
| PI567350A | Shu pi huang dou | China | VI | 207 | 146 | 66 | 61 | 1 | 66 |
| PI567378 | Ba yue zha | China | VI | 135 | 193 | 17 | 24 | 67 | 67 |
| Fendou78 | - | China | IV | 11 | 93 | 14 | 68 | 207 | 68 |
| PI341264 | - | Liberia | VI | 68 | 31 | 173 | 105 | 59 | 68 |
| PI171443 | Tea-bean | China | VI | 147 | 69 | 121 | 67 | 8 | 69 |
| PI592939 | Jin dou 16 | China | IV | 6 | 3 | 106 | 70 | 186 | 70 |
| PI567758 | Pei xian tu shan da ping ding huang | China | IV | 48 | 72 | 2 | 134 | 94 | 72 |
| PI462312 | Ankur | India | VIII | 95 | 19 | 64 | 74 | 83 | 74 |
| PI639575 | - | Burundi | VIII | 134 | 177 | 62 | 50 | 74 | 74 |
| PI330635 | - | South Africa | VII | 206 | 12 | 182 | 48 | 74 | 74 |
| PI322691 | Jubiltan 109 | Mozambique | IX | 167 | 44 | 74 | 144 | 33 | 74 |
| PI574483 | Jin dou No. 5 | China | IV | 57 | 75 | 10 | 97 | 200 | 75 |
| PI574484 | Jin dou No. 6 | China | IV | 2 | 176 | 20 | 76 | 208 | 76 |
| PI486330 | Macs-75 | India | VIII | 43 | 84 | 68 | 160 | 76 | 76 |
| PI434981 | Indo 226 | Central African Republic | VIII | 76 | 87 | 117 | 7 | 70 | 76 |
| PI330634 | - | South Africa | VII | 55 | 179 | 119 | 73 | 76 | 76 |
| PI548657 | Jackson | United States | VII | 77 | 153 | 44 | 205 | 13 | 77 |
| PI603566 | Jin dou No. 4 | China | III | 20 | 100 | 46 | 78 | 200 | 78 |
| PI574485 | Jin dou No. 9 | China | IV | 46 | 100 | 76 | 78 | 208 | 78 |
| PI567345 | Niu mao huang | China | VI | 78 | 52 | 166 | 152 | 11 | 78 |
| PI567295 | Bian huang dou | China | VIII | 154 | 164 | 78 | 19 | 2 | 78 |
| PI639576 | - | Burundi | VIII | 79 | 107 | 34 | 65 | 174 | 79 |
| PI210349 | Jubiltan 65 | Mozambique | VIII | 65 | 139 | 79 | 28 | 162 | 79 |
| PI603537D | (Niu yan jing quan zi) | China | VII | 89 | 41 | 80 | 140 | 28 | 80 |
| PI603517A | Lao shu pi | China | VI | 25 | 77 | 135 | 81 | 137 | 81 |
| PI429330 | - | Nigeria | VIII | 93 | 130 | 54 | 81 | 53 | 81 |
| PI639573 | - | Burundi | VIII | 199 | 25 | 163 | 51 | 81 | 81 |
| PI417561 | 48.S.103 DL/63/180 | South Africa | VI | 75 | 32 | 83 | 114 | 123 | 83 |
| Fendou56 | - | China | IV | 9 | 30 | 98 | 149 | 83 | 83 |
| PI603535 | Hei zong huang dou | China | VIII | 17 | 156 | 192 | 83 | 83 | 83 |
| PI376069 | DRO 9 | Cameroon | VIII | 184 | 10 | 159 | 36 | 83 | 83 |
| PI553046 | Gasoy 17 | United States | VII | 83 | 175 | 72 | 120 | 13 | 83 |
| PI269518B | (Koolat) | Pakistan | VI | 84 | 174 | 71 | 26 | 196 | 84 |
| PI381680 | S7 | Uganda | VII | 85 | 23 | 188 | 88 | 67 | 85 |
| PI603521 | Huang dou | China | VIII | 62 | 190 | 196 | 85 | 83 | 85 |
| PI322692 | Max C.P1159A8 | Australia | IX | 53 | 86 | 123 | 21 | 157 | 86 |
| PI346300 | - | India | VII | 30 | 192 | 86 | 88 | 49 | 86 |
| PI567683B | (Zheng zhou niu yao qi) | China | VI | 157 | 88 | 15 | 92 | 73 | 88 |
| PI603506 | Xiao ke zao huang dou | China | VI | 90 | 136 | 89 | 16 | 33 | 89 |
| PI548975 | Centennial | United States | VI | 56 | 39 | 90 | 198 | 95 | 90 |
| PI221715 | - | South Africa | VII | 74 | 90 | 162 | 111 | 13 | 90 |
| PI567403A | Shuan huang dou | China | VII | 156 | 26 | 139 | 90 | 62 | 90 |
| PI381657 | 3H55 F4/9/2 | Uganda | VIII | 158 | 45 | 92 | 77 | 122 | 92 |
| PI595645 | Benning | United States | VII | 91 | 92 | 39 | 184 | 192 | 92 |
| PI578494A | Jin dou No. 1 | China | IV | 46 | 58 | 94 | 142 | 208 | 94 |
| Fendou65 | - | China | IV | 1 | 94 | 53 | 104 | 147 | 94 |
| Jindou19 | - | China | IV | 3 | 95 | 30 | 180 | 205 | 95 |
| PI522236 | Thomas | United States | VII | 96 | 73 | 42 | 190 | 95 | 95 |
| PI567315 | Hong huang dou | China | VII | 27 | 7 | 111 | 156 | 95 | 95 |
| PI323570 | - | India | VII | 97 | 126 | 11 | 164 | 76 | 97 |
| PI429328 | - | Nigeria | VIII | 21 | 98 | 32 | 126 | 151 | 98 |
| PI567412 | Yi wo feng | China | VI | 198 | 105 | 8 | 98 | 2 | 98 |
| PI567405 | Wei zi dou | China | VI | 60 | 99 | 118 | 43 | 108 | 99 |
| PI555453 | Hagood | United States | VII | 99 | 150 | 87 | 171 | 37 | 99 |
| PI159096 | 41S77 | South Africa | VII | 197 | 97 | 170 | 99 | 39 | 99 |
| PI639574 | - | Burundi | VIII | 115 | 66 | 93 | 115 | 101 | 101 |
| FC003659 | Da Wu Don | China | VI | 101 | 68 | 193 | 147 | 11 | 101 |
| PI567349B | (Shu pi huang dou) | China | VI | 70 | 197 | 101 | 150 | 27 | 101 |
| PI497967 | - | India | VII | 151 | 205 | 13 | 101 | 13 | 101 |
| PI482601 | - | Zimbabwe | IX | 173 | 129 | 95 | 85 | 102 | 102 |
| PI567332 | Huo huang dou | China | VI | 192 | 201 | 70 | 102 | 63 | 102 |
| PI567404B | (Wang shan hou) | China | VI | 200 | 2 | 102 | 158 | 6 | 102 |
| PI639572 | - | Ghana | VIII | 44 | 74 | 114 | 103 | 185 | 103 |
| PI341242 | Hernon 247 | Tanzania | IX | 104 | 103 | 146 | 112 | 50 | 104 |
| PI648270 | Osage | United States | V | 155 | 85 | 104 | 203 | 95 | 104 |
| PI567394A | Jiu yue han | China | VI | 105 | 108 | 190 | 41 | 33 | 105 |
| PI567207 | - | Georgia | VI | 127 | 102 | 107 | 40 | 178 | 107 |
| PI089775 | - | China | VI | 164 | 42 | 169 | 66 | 107 | 107 |
| PI603538C | (Wan dou zao) | China | VIII | 108 | 70 | 177 | 60 | 137 | 108 |
| PI381661 | Bukalasa 6 | Uganda | VIII | 144 | 80 | 16 | 108 | 179 | 108 |
| PI090406 | - | China | VI | 133 | 24 | 81 | 109 | 128 | 109 |
| PI381683 | S36 | Uganda | VI | 153 | 6 | 50 | 173 | 109 | 109 |
| PI322695 | Bicolor do Cuima | Angola | VI | 128 | 159 | 99 | 44 | 109 | 109 |
| PI341253 | CMS | Sudan | IX | 110 | 157 | 197 | 91 | 93 | 110 |
| PI430736 | Kudu | Zimbabwe | VI | 160 | 55 | 110 | 165 | 81 | 110 |
| PI508266 | Young | United States | VI | 40 | 189 | 111 | 130 | 42 | 111 |
| PI553045 | Cook | United States | VIII | 28 | 169 | 113 | 179 | 106 | 113 |
| PI567377B | (Ba yue zha) | China | VI | 209 | 196 | 1 | 113 | 8 | 113 |
| PI341241A | Seminole | Israel | IX | 114 | 118 | 24 | 69 | 182 | 114 |
| PI615694 | N7001 | United States | VII | 23 | 47 | 204 | 186 | 114 | 114 |
| WOODRUFF | - | United States | VII | 16 | 49 | 179 | 194 | 114 | 114 |
| G00-3213 | - | United States | VII | 42 | 49 | 179 | 172 | 114 | 114 |
| PI602597 | Boggs | United States | VI | 140 | 67 | 59 | 200 | 114 | 114 |
| PI548659 | Braxton | United States | VII | 73 | 169 | 42 | 190 | 114 | 114 |
| PI548989 | Ransom | United States | VII | 15 | 78 | 150 | 195 | 114 | 114 |
| PI548660 | Bragg | United States | VII | 102 | 115 | 72 | 192 | 114 | 114 |
| PI341241B | (Seminole) | Israel | IX | 109 | 162 | 115 | 53 | 146 | 115 |
| PI553039 | Davis | United States | VI | 116 | 155 | 82 | 154 | 112 | 116 |
| PI438430 | - | Israel | VII | 52 | 141 | 116 | 187 | 21 | 116 |
| PI567493 | Huang dou | China | IV | 190 | 116 | 7 | 176 | 56 | 116 |
| PI603519 | Lu da dou | China | VI | 34 | 117 | 142 | 115 | 151 | 117 |
| PI567206 | GL2674/90 | Georgia | VI | 119 | 132 | 91 | 160 | 44 | 119 |
| PI212605 | - | Afghanistan | VI | 120 | 191 | 57 | 38 | 196 | 120 |
| PI374219 | Blyvoor | South Africa | VI | 187 | 48 | 120 | 17 | 149 | 120 |
| PI567329 | Huang huang dou | China | VI | 88 | 120 | 149 | 145 | 44 | 120 |
| PI170890 | - | South Africa | VI | 121 | 45 | 176 | 14 | 203 | 121 |
| PI428691 | - | India | VIII | 138 | 121 | 88 | 160 | 23 | 121 |
| PI306702A | 3H/1 | Kenya | IX | 126 | 18 | 9 | 122 | 163 | 122 |
| PI221716 | - | South Africa | VII | 122 | 134 | 75 | 177 | 76 | 122 |
| PI416937 | Houjaku Kuwazu | Japan | VI | 69 | 15 | 189 | 146 | 123 | 123 |
| PI603529 | Hei huang dou | China | VIII | 112 | 123 | 27 | 206 | 151 | 123 |
| NTCPR94-5157 | - | United States | VI | 7 | 124 | 153 | 121 | 187 | 124 |
| PI374221 | Welkom | South Africa | VI | 195 | 34 | 198 | 124 | 76 | 124 |
| N04-9646 | - | United States | VII | 82 | 81 | 128 | 125 | 141 | 125 |
| N06-7194 | - | United States | VIII | 31 | 133 | 125 | 183 | 111 | 125 |
| PI324067 | Hernon 237 | Zimbabwe | VII | 189 | 109 | 126 | 85 | 126 | 126 |
| PI381662 | Hernon 49 | Uganda | VI | 180 | 65 | 127 | 35 | 177 | 127 |
| PI090768 | - | China | VI | 172 | 91 | 96 | 202 | 127 | 127 |
| PI458517 | Xiao Wuyie | China | III | 98 | 148 | 21 | 127 | 130 | 127 |
| N05-7432 | - | United States | VIII | 8 | 79 | 143 | 128 | 134 | 128 |
| PI603540A | Hei huang dou | China | VII | 50 | 37 | 185 | 129 | 172 | 129 |
| PI398276 | Chirpan 90 (Bulgaria) | South Korea | IV | 129 | 71 | 35 | 169 | 174 | 129 |
| PI603534B | (Da niu mao huang) | China | VIII | 12 | 204 | 165 | 49 | 129 | 129 |
| N06-7543 | - | United States | VII | 59 | 114 | 129 | 181 | 164 | 129 |
| PI171441 | Mud-bean | China | VI | 202 | 199 | 45 | 6 | 131 | 131 |
| PI603539A | Huang dou | China | VI | 107 | 161 | 154 | 115 | 131 | 131 |
| PI221717 | - | South Africa | VI | 146 | 131 | 51 | 173 | 114 | 131 |
| PI265498 | - | Zaire | VIII | 131 | 166 | 148 | 55 | 83 | 131 |
| PI341245 | Avoyelles | Tanzania | IX | 150 | 185 | 132 | 33 | 99 | 132 |
| PI603509 | Huang dou | China | VIII | 81 | 200 | 200 | 132 | 83 | 132 |
| PI567410A | Yang huang dou | China | VII | 179 | 184 | 133 | 58 | 99 | 133 |
| PI247678 | Herman | Zaire | VIII | 143 | 149 | 97 | 9 | 135 | 135 |
| PI548656 | Lee | United States | VI | 58 | 172 | 131 | 135 | 164 | 135 |
| PI532458 | Ba yue bao | China | VIII | 18 | 135 | 161 | 175 | 125 | 135 |
| PI531068 | Stonewall | United States | VII | 67 | 22 | 136 | 199 | 206 | 136 |
| PI567350B | (Shu pi huang dou) | China | VI | 136 | 111 | 160 | 148 | 24 | 136 |
| PI279081 | Masterpiece | South Africa | VII | 204 | 76 | 137 | 3 | 203 | 137 |
| PI210350 | Jubiltan 67 | Mozambique | IX | 183 | 137 | 47 | 94 | 151 | 137 |
| PI603528 | Hei ke zha | China | VII | 14 | 187 | 195 | 137 | 28 | 137 |
| PI603536 | Hui huang dou | China | VIII | 19 | 171 | 130 | 139 | 151 | 139 |
| PI592937 | Jin dou 14 | China | IV | 169 | 140 | 19 | 25 | 158 | 140 |
| PI159095 | 41S31 | South Africa | VII | 141 | 14 | 172 | 99 | 172 | 141 |
| PI567393 | Jiu yue han | China | VII | 170 | 167 | 141 | 131 | 70 | 141 |
| PI567314 | Hei you huang dou | China | VI | 142 | 206 | 52 | 95 | 179 | 142 |
| PI505649B | - | Zambia | IX | 205 | 202 | 25 | 115 | 142 | 142 |
| PI417562 | 54.S.30 DL/64/185 | South Africa | VI | 208 | 63 | 184 | 138 | 142 | 142 |
| PI603532 | Hong li huang dou | China | VI | 159 | 144 | 63 | 115 | 196 | 144 |
| PI567205 | GL2671/89 | Georgia | VI | 161 | 83 | 145 | 39 | 164 | 145 |
| PI556949 | Ke feng No. 1 | China | IV | 193 | 173 | 3 | 12 | 147 | 147 |
| PI641156 | NC-Raleigh | United States | VII | 87 | 180 | 147 | 195 | 56 | 147 |
| PI495016 | Nuwara Eliya Local | Sri Lanka | IX | 148 | 43 | 158 | 33 | 191 | 148 |
| PI612157 | Prichard | United States | VIII | 149 | 122 | 100 | 188 | 164 | 149 |
| PI592756 | Dillon | United States | VI | 99 | 150 | 84 | 177 | 160 | 150 |
| PI494851 | - | Zambia | VI | 162 | 150 | 61 | 154 | 112 | 150 |
| PI603520 | Huang dou | China | VI | 118 | 181 | 154 | 151 | 8 | 151 |
| PI090499 | Black and white | China | VI | 152 | 28 | 6 | 197 | 188 | 152 |
| PI205384 | - | Pakistan | VI | 94 | 96 | 156 | 153 | 194 | 153 |
| PI603514 | Ni ba dou | China | VI | 103 | 165 | 157 | 170 | 37 | 157 |
| PI424131 | Buffalo | Zimbabwe | VII | 191 | 158 | 185 | 136 | 102 | 158 |
| PI561375 | Qi huang No. 1 | China | V | 186 | 163 | 4 | 133 | 159 | 159 |
| PI617045 | NC-Roy | United States | VI | 86 | 145 | 202 | 159 | 164 | 159 |
| PI518664 | Hutcheson | United States | V | 92 | 160 | 28 | 184 | 192 | 160 |
| PI145079 | Hernon No. 6 | Zimbabwe | VII | 163 | 17 | 168 | 201 | 44 | 163 |
| PI647085 | N7002 | United States | VII | 13 | 128 | 194 | 168 | 164 | 164 |
| PI323278 | K-30 | Pakistan | IX | 165 | 188 | 167 | 47 | 105 | 165 |
| PI603534A | Da niu mao huang | China | VII | 32 | 195 | 203 | 166 | 83 | 166 |
| PI159094 | 35S377 | South Africa | VII | 196 | 112 | 171 | 167 | 151 | 167 |
| PI574486 | Jin dou 13 | China | III | 168 | 178 | 76 | 78 | 190 | 168 |
| PI221714 | - | South Africa | VI | 171 | 168 | 60 | 203 | 13 | 168 |
| PI170886 | - | South Africa | VI | 174 | 16 | 174 | 13 | 188 | 174 |
| PI603513A | Xiao niu mao huang | China | VIII | 22 | 203 | 199 | 32 | 182 | 182 |
| NCC06-1090 | - | United States | VI | 182 | 186 | 151 | 182 | 136 | 182 |
| PI599333 | Musen | United States | VI | 188 | 183 | 103 | 188 | 164 | 183 |

^a^ Canopy wilting data are from Steketee et al. (2018)

^b^ Breeding values were calculated for each trait within an individual environment, and then summed across environments. These summed breeding values for each trait were ranked in ascending (canopy wilting, nitrogen concentration, δ^15^N, and NDTR to AgNO_3_) or descending (δ^13^C) order based on whether negative or positive breeding values would be more favorable for the trait.
