# Supplementary material for: Unraveling the genetic architecture for carbon and nitrogen related traits and leaf hydraulic conductance in soybean using genome-wide association analyses
Source: BMC Genomics. 2019 Nov 6;20:811. doi: 10.1186/s12864-019-6170-7 (PMC6836393; doi:10.1186/s12864-019-6170-7)
Supplement: Supplementary file 2 — Additional file 2. Genome-wide Manhattan plots for carbon and nitrogen related traits. [file 12864_2019_6170_MOESM2_ESM.docx]

**Additional file 2**


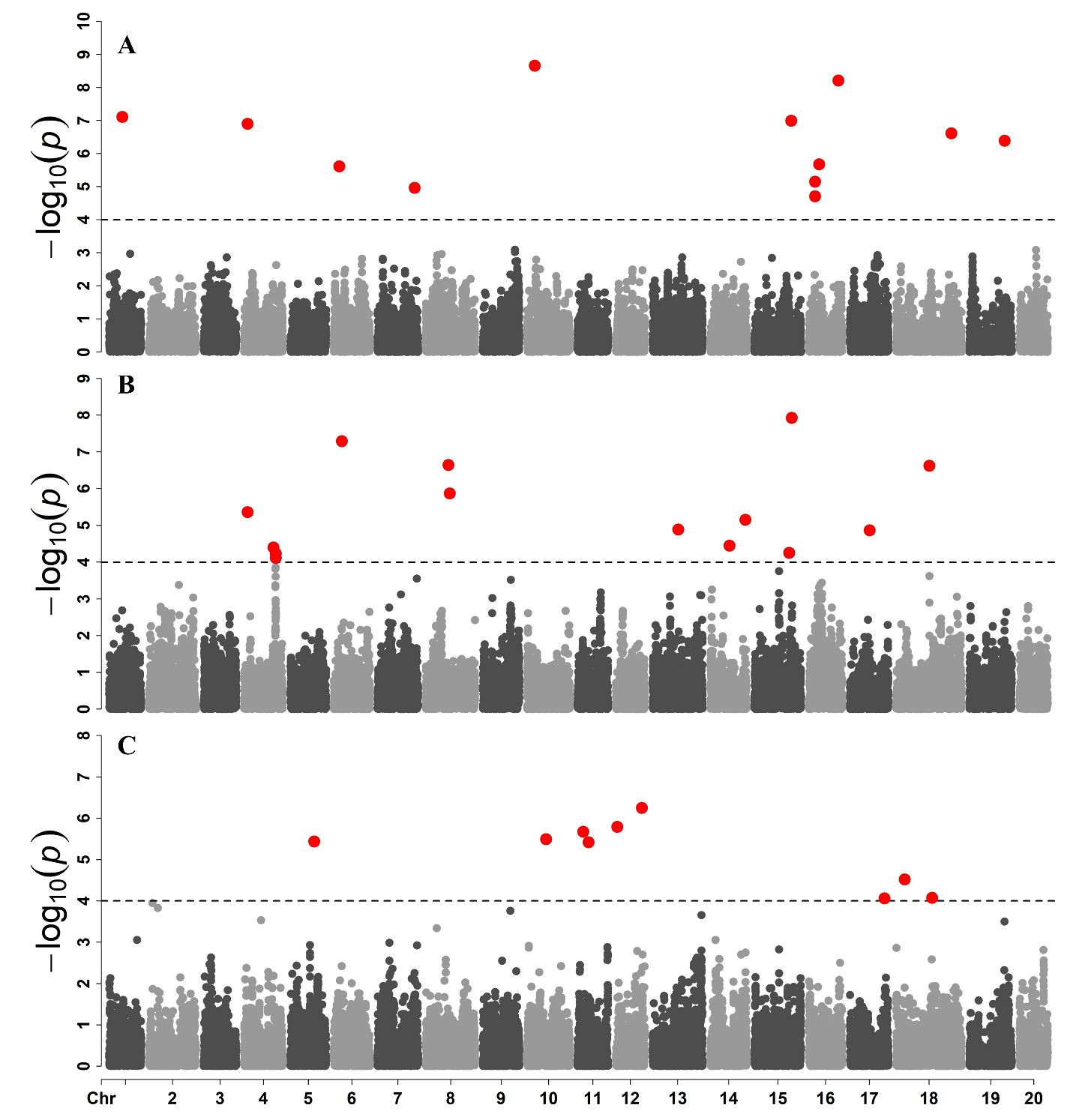


Manhattan plots for carbon isotope composition (δ^13^C) for A) Both, B) GA-15, and C) GA-16 environments. The X-axis is the genomic position of SNPs across the soybean genome by chromosome, and the Y-axis is the -log_10_ of the p-values obtained from the GWAS model. SNPs that were above significance threshold (-log_10_*(P)* > 4) are colored in red and enlarged.


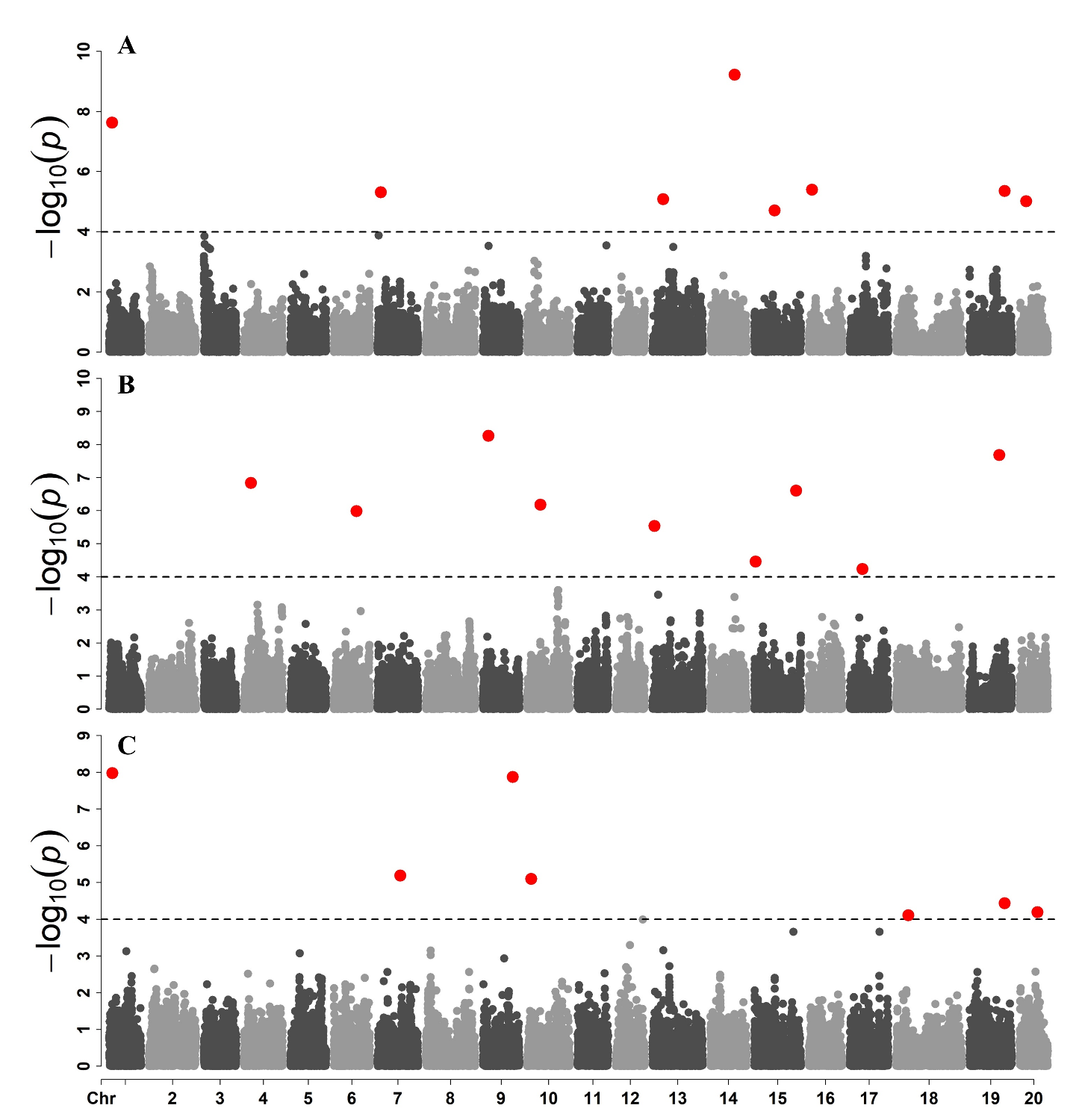


Manhattan plots for nitrogen isotope composition (δ^15^N) for A) Both, B) GA-15, and C) GA-16 environments. The X-axis is the genomic position of SNPs across the soybean genome by chromosome, and the Y-axis is the -log_10_ of the p-values obtained from the GWAS model. SNPs that were above significance threshold (-log_10_*(P)* > 4) are colored in red and enlarged.


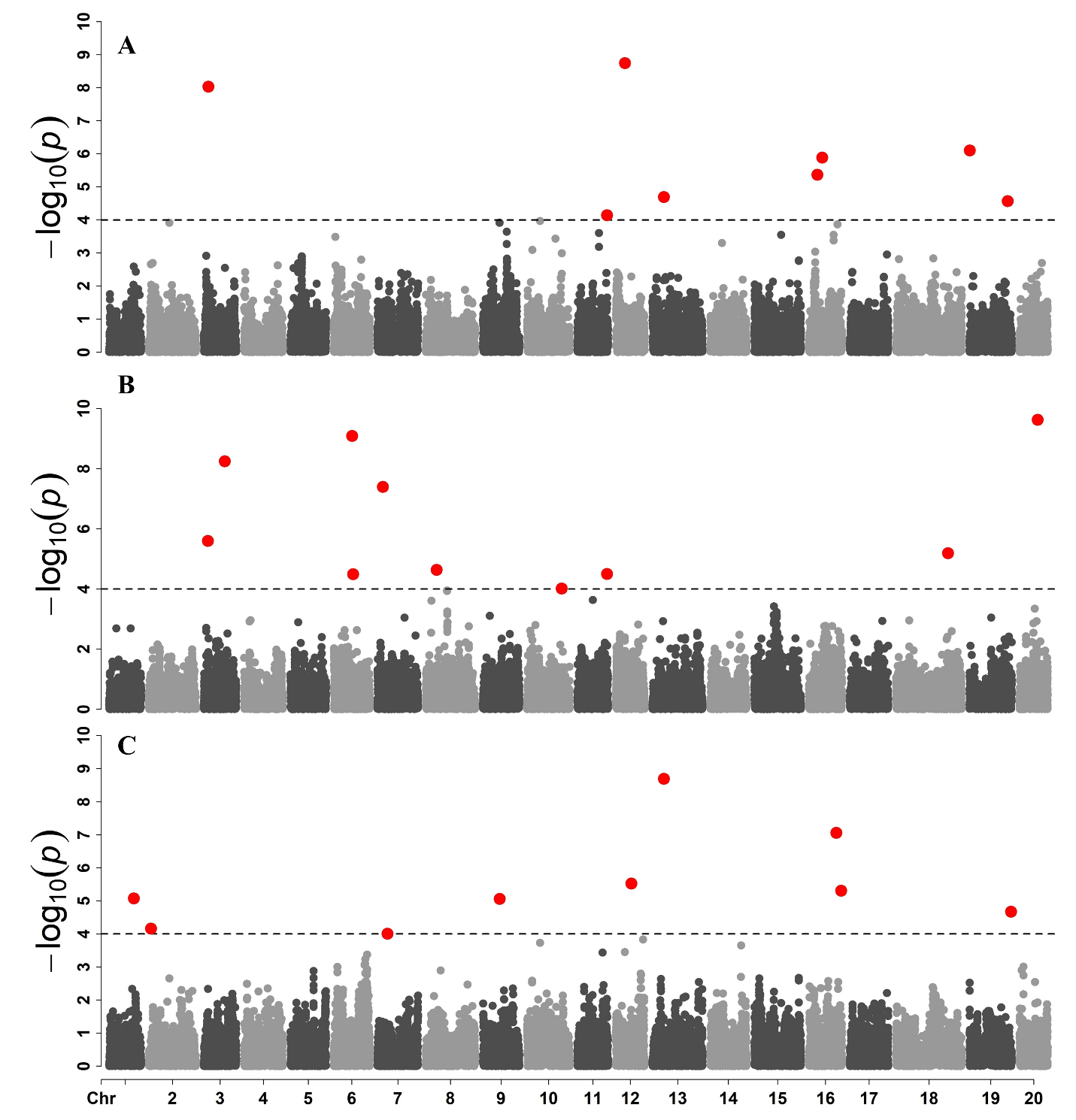


Manhattan plots for nitrogen concentration for A) Both, B) GA-15, and C) GA-16 environments. The X-axis is the genomic position of SNPs across the soybean genome by chromosome, and the Y-axis is the -log_10_ of the p-values obtained from the GWAS model. SNPs that were above significance threshold (-log_10_*(P)* > 4) are colored in red and enlarged.
