# Supplementary material for: Unraveling the genetic architecture for carbon and nitrogen related traits and leaf hydraulic conductance in soybean using genome-wide association analyses
Source: BMC Genomics. 2019 Nov 6;20:811. doi: 10.1186/s12864-019-6170-7 (PMC6836393; doi:10.1186/s12864-019-6170-7)
Supplement: Supplementary file 3 — Additional file 3. Candidate genes and their functional annotation identified using the Glyma2.1 gene models in SoyBase within plus or minus 10 kb of SNPs significantly associated with carbon and nitrogen related traits. [file 12864_2019_6170_MOESM3_ESM.docx]

**Additional file 3**

Candidate genes and their functional annotation identified using the Glyma2.1 gene models in SoyBase (www.soybase.org) within plus or minus 10 kb of the most significant SNP for carbon and nitrogen related traits from GWAS in each environment and using across environments data.

| Carbon Isotope Composition | | | | |
| --- | --- | --- | --- | --- |
| Locus | SNP | Gene Name | Annotation | GO Terms |
| 11 | ss715607234 | Glyma.10g047500 | Protein phosphatase 2C family protein | Protein dephosphorylation; catalytic activity; hydrolase activity; cation binding; metal ion binding |
|  |  | Glyma.10g047600 | Integral membrane TerC family protein | Membrane; integral component of membrane |
| 16 | ss715612828 | Glyma.12g221000 | Peroxisomal membrane 22 kDa (Mpv17/PMP22) family protein | Cytoplasm; integral component of membrane |
|  |  | Glyma.12g221100 | Purple acid phosphatase 10 | Dephosphorylation; acid phosphatase activity; hydrolase activity; metal ion binding |
|  |  | Glyma.12g221200 | N/A | N/A |
|  |  | Glyma.12g221300 | N/A | N/A |
| 22 | ss715622149 | Glyma.15g248200 | Ubiquitin-specific protease 15 | Ubiquitin-dependent protein catabolic process; protein deubiquitination; membrane; integral component of membrane; ubiquitin thiolesterase activity; metal ion binding |
|  |  | Glyma.15g248300 | SOUL heme-binding family protein | N/A |
|  |  |  |  |  |
| Nitrogen Isotope Composition | | | | |
| Locus | SNP | Gene Name | Annotation | GO Terms |
| 2 | ss715578694 | Glyma.01g021000 | Elicitor-activated gene 3-2 | Oxidation-reduction process; zinc ion binding; oxidoreductase activity; metal ion binding |
| 7 | ss715603834 | Glyma.09g043700 | 3-ketoacyl-CoA synthase 21 | Fatty acid biosynthetic process; metabolic process; lipid biosynthetic process; membrane; integral component of membrane; catalytic activity; transferase activity |
|  |  | Glyma.09g043800 | Pleiotropic regulatory locus 1 | Protein binding |
|  |  | Glyma.09g043900 | Transducin/WD40 repeat-like superfamily protein | Signal transduction; binding; protein binding |
| 13 | ss715618124 | Glyma.14g144700 | Amino acid permease 8 | Amino acid transmembrane transport; plasma membrane; membrane; integral component of membrane; amino acid transmembrane transporter activity |
|  |  | Glyma.14g144800 | N/A | N/A |
|  |  |  |  |  |
| Nitrogen Content | | | | |
| Locus | SNP | Gene Name | Annotation | GO Terms |
| 15 | ss715613118 | Glyma.12g065700 | N/A | N/A |
|  |  | Glyma.12g065800 | C2H2-type zinc finger family protein | Nucleic acid binding |
|  |  | Glyma.12g065900 | N/A | N/A |
| 17 | ss715616699 | Glyma.13g070200 | Polynucleotidyl transferase, ribonuclease H-like superfamily protein | RNA phosphodiester bond hydrolysis; nucleic acid binding; RNA-DNA hybrid ribonuclease activity |
|  |  | Glyma.13g070300 | Cellulose-synthase-like C4 | Membrane; integral component of membrane; transferase activity; transferring glycosyl groups |
| 26 | ss715638016 | Glyma.20g153600 | Phosphoglucomutase | Carbohydrate metabolic process; glycogen biosynthetic process; glucose metabolic process; detection of gravity; starch biosynthetic process; galactose catabolic process; organic substance metabolic process; cytosol; chloroplast stroma; stromule; magnesium ion binding; phosphoglucomutase activity; intramolecular transferase activity; metal ion binding |
|  |  | Glyma.20g153700 | K-box region and MADS-box transcription factor family protein | Transcription, DNA-templated; regulation of transcription; positive regulation of transcription by RNA polymerase II; nucleus; DNA binding; DNA binding transcription factor activity; protein dimerization activity |
